# A Guide to Signal Processing Algorithms for Nanopore Sensors

**DOI:** 10.1021/acssensors.1c01618

**Published:** 2021-10-04

**Authors:** Chenyu Wen, Dario Dematties, Shi-Li Zhang

**Affiliations:** ‡ Division of Solid-State Electronics, Department of Electrical Engineering, Uppsala University, SE-751 03 Uppsala, Sweden; ¶ Instituto de Ciencias Humanas, Sociales y Ambientales, CONICET Mendoza Technological Scientific Center, Mendoza M5500, Argentina

**Keywords:** nanopore sensing, signal processing algorithm, pulse-like signal, spike recognition, feature extraction, analyte identification, machine learning, neural network

## Abstract

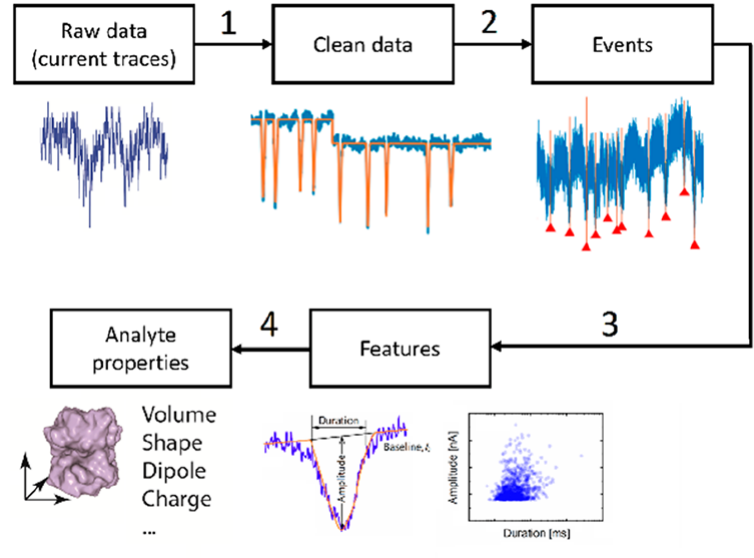

Nanopore technology holds great promise for a wide range of applications such as biomedical sensing, chemical detection, desalination, and energy conversion. For sensing performed in electrolytes in particular, abundant information about the translocating analytes is hidden in the fluctuating monitoring ionic current contributed from interactions between the analytes and the nanopore. Such ionic currents are inevitably affected by noise; hence, signal processing is an inseparable component of sensing in order to identify the hidden features in the signals and to analyze them. This Guide starts from untangling the signal processing flow and categorizing the various algorithms developed to extracting the useful information. By sorting the algorithms under Machine Learning (ML)-based versus non-ML-based, their underlying architectures and properties are systematically evaluated. For each category, the development tactics and features of the algorithms with implementation examples are discussed by referring to their common signal processing flow graphically summarized in a chart and by highlighting their key issues tabulated for clear comparison. How to get started with building up an ML-based algorithm is subsequently presented. The specific properties of the ML-based algorithms are then discussed in terms of learning strategy, performance evaluation, experimental repeatability and reliability, data preparation, and data utilization strategy. This Guide is concluded by outlining strategies and considerations for prospect algorithms.

Nanopore sensors have been developed for decades to target multiple applications, including DNA sequencing,^1^ protein profiling,^2^ small chemical molecule detection,^3,4^ and nanoparticle characterization.^5,6^ Nanopore sensor is inspired by the Coulter cell counter^7^ and realizes a task by matching its dimension to that of analytes, molecules or nanoparticles. Thus, it possesses an extremely succinct structure, a nanoscale pore in an ultrathin membrane. Its sensing function is based on a simple working principle: the passage of an analyte temporarily blocks a size-proportional volume of the pore and induces a spike signal on the monitoring ionic current at a given bias voltage. Information about passing analytes is hidden in the corresponding current spikes, i.e., translocation spikes distributed on the ionic current traces. By processing the signal and analyzing the features of the spikes such as amplitude, width (duration), occurrence frequency, and waveform, the properties of the analytes can be inferred, including size, shape, charge, dipole moment, and concentration. Therefore, signal processing is the crucial link to interpreting the signal by assigning the associated features to relevant physical properties. In general, signal processing comprises denoising, spike recognition, feature extraction, and analysis. A powerful signal processing algorithm should be able to isolate signals from a noisy background, extract useful information, and utilize the multidimensional information synthetically to accurately derive the properties of the analytes.

Low-pass filters have been adopted as a simple approach to removing the background noise. However, this function risks filtering out the important high-frequency components naturally present in signals representing rapid changes of ionic current associated with translocation spikes that carry informative waveform details related to the target analytes. Thus, self-adaptive filters and advanced current level tracing algorithms have been developed.^8,9^ Traditional algorithms are mainly based on a user-defined amplitude threshold as a criterion for detection of translocation spikes. Apparently, the choice of this threshold determines how successful a spike is singled out and how good the quality of the subsequent feature extraction is. However, the threshold is usually chosen based on the experience of individuals dealing with the data. It is, hence, a subjective process. Moreover, using the extracted features to infer the properties of the analytes relies mainly on physical models that build upon a comprehensive understanding of the physiochemical process involved in the translocation. Unfortunately, generalized models and algorithms for this purpose are yet to be developed.

Concurrently, Machine Learning (ML) has revolutionized the signal processing landscape. In this regard, ML algorithms for nanopore sensing have seen rapid advancements in noise mitigation, spike recognition, feature extraction, and analyte classification. The learning process usually demands a huge number of well-labeled data sets, which is challenging. Furthermore, the applicability of ML-based algorithms is restricted by the accessibility of training data sets. In addition, ML-based algorithms usually work as a black box so that a user has limited knowledge of their operation.^10^ This shortcoming can impair the control and usage of the algorithms and further adversely affect the interpretation of the results. Combining ML-based algorithms with physics-based models to exert respective advantages is considered a promising approach to attaining high-fidelity signal processing.

Reviews on processing the signals from nanopore sensors are sparse in the literature despite their scientific relevance and technological potential. One of the few reviews on signal processing technologies for identification of nanopore biomolecule includes both software algorithms and hardware readout circuits/systems.^11^ A more general topic on ML-based algorithms for signals from biosensors touches upon nanopore sensing.^12^ In addition, mini-reviews on some specific issues of signal processing for nanopore sensors and related sensors can be found, such as ML for identification of single biomolecules,^13^ virus detection,^14^ and nanopore electrochemistry.^15^ Concomitantly, signal processing algorithms for nanopore sensing have been rapidly developed by adopting various strategies and techniques. It is, therefore, ripe to request a systematic treatment of the different algorithms, including both non-ML-based and ML-based, with respect to their architectures and properties. This Guide offers a general description of the explored signal processing algorithms for nanopore sensors and, thereafter, proposes guidelines for the development of prospect algorithms.

The Guide starts by categorizing the reported algorithms as non-ML type and ML type. Each category is generalized under the umbrella of a common signal processing flow to guide the discussion of specific algorithms in terms of development tactics and features. The focus will then be placed on the ML-based algorithms by scrutinizing the respective strategies and properties. Specifically, the discussion spans learning strategy, performance evaluation, experimental repeatability and reliability, data quality, data preparation, and data utilization. The discussion also concerns challenges, possible solutions, and special considerations for nanopore signals. Finally, strategies and considerations are outlined for prospect algorithms to conclude this Guide.

## Signal Processing Flow

**Figure 1:**
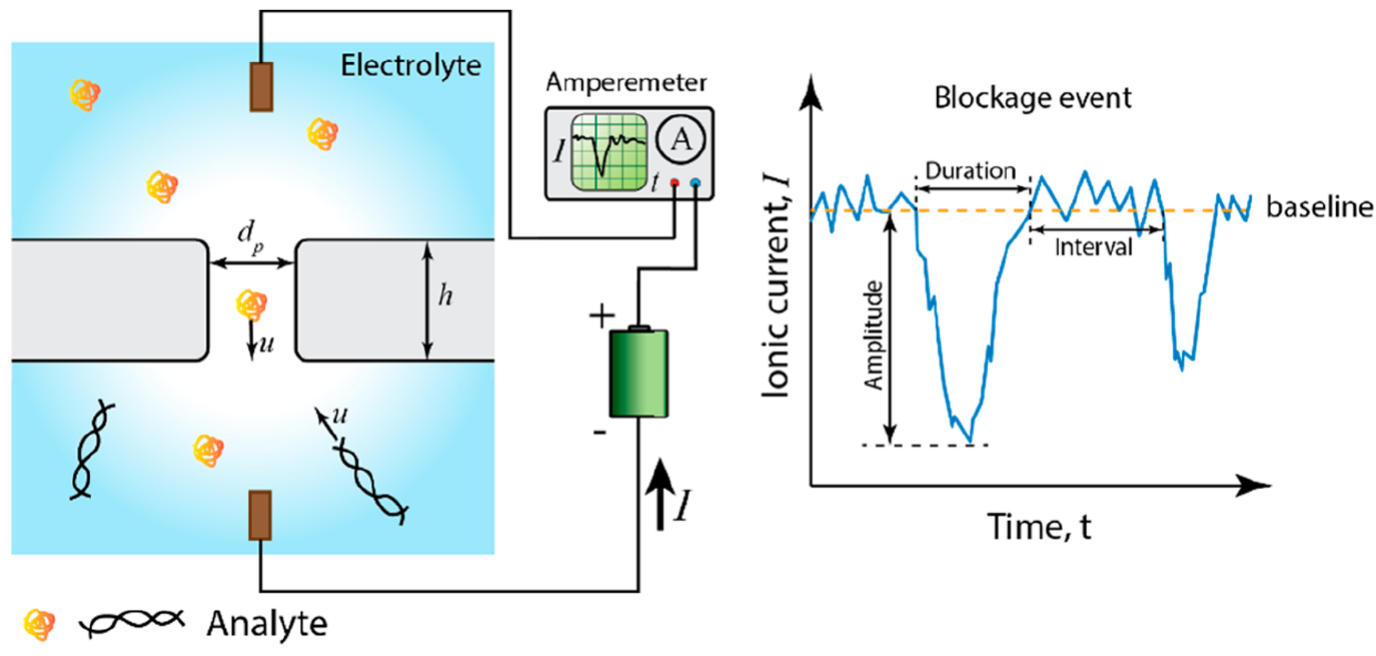
Schematics showing the device structure of a typical nanopore sensor (left) and a typical current trace with spikes generated by analyte translocations (right).

The nanopore device used for sensing is usually immersed in an electrolyte, as shown in the left panel of Figure 1. The membrane embedding a nanopore separates the electrolyte into two compartments. The only electrical connection between them is the nanopore. By applying a bias voltage across the membrane, a steady ionic current, named open-pore current, is generated, which constitutes the baseline of the signal. The electric field also drives charged analytes dispersed in the electrolyte to pass through the nanopore. During the translocation, the analytes temporarily block a certain volume of the pore, proportional to their size. Such blockages usually cause spike-like current variations, as seen in the right panel of Figure 1, that are of central interest for signal processing. The ionic current is anticipated to resume the open-pore level once the translocations complete. Other designs can also be adopted to generate signals for nanopore sensing. For example, functionalizing the nanopore surface with a probe molecule can generate a specific interaction with target analytes resulting in characteristic signals on the monitoring ionic current trace.^16^ Such signals arising from specific interactions,^17^ adsorption–desorption processes,^18^ clogging,^19^ nanopore morphology changes,^20^ and open–close activities of channels^21^ can also be dealt with in the same framework designed for processing the translocation-caused spike signals.

**Figure 2:**
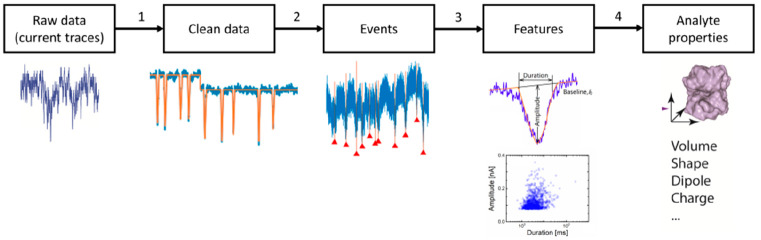
Typical signal processing flow for nanopore sensors.

The typical signal processing flow for nanopore sensors is summarized in Figure 2. Raw data here refer to those directly acquired experimentally and background noise is persistently present, while clean data represent those after the denoising process with which the background noise is sufficiently mitigated. With raw data at hand, a complete signal processing scheme comprises four consecutive steps as follows.

Step 1 Denoise raw data to generate clean data, typically via low-pass filters in the frequency domain. This step can be omitted if the quality of the raw data, i.e., signal-to-noise ratio, is acceptable.
Step 2 Identify and extract translocation events represented as spikes on current traces, frequently based on a user-defined threshold of the amplitude as a criterion to separate a true translocation-generated spike from the noise fluctuation.
Step 3 Extract features of these spikes based on various methods such as physical models, peak analysis algorithms, and algorithms of feature analysis in the frequency domain.
Step 4 Infer the properties of the translocating analytes from the extracted features.

In general, the parameters/structures of the ML-based algorithms can be dynamically adjusted in the training process according to the input data in order to achieve an improved performance toward the goal.^22^ For a typical ML algorithm, the input data is usually deliberately divided into a training data set and a test data set. An automatic adjustment of the parameters/structures only applies to the training data set.^22^ However, the implicit differentiation of the training and test data sets is not a necessity. For example, an ML algorithm can adjust its parameters/structures upon processing each and every input. Furthermore, the input data can be labeled or unlabeled so that the associated algorithms are based on supervised or unsupervised learning, respectively.

An algorithm can be regarded as ML-based if its current output is associated with its historical input or distribution of input, i.e., it “learns” from the history/distribution and exploits the hidden relations/patterns carried in the input data. Such learning can be explicit, as in a supervised training process for algorithms with labeled data sets. Nevertheless, the learning can also be implicit, as in some unsupervised clustering algorithms with a learning-by-doing manner. Therefore, an ML-based algorithm always relates to tunable weights, adjustable architectures, self-adaptable parameters, memory, etc. In contrast, a non-ML algorithm usually outputs in real time, i.e., it records no history data and, hence, its current output/systematic state is not influenced by any such history/input distribution. However, the boundary between non-ML and ML algorithms is not always sharp and clear. For example, algorithms for spike recognition and baseline tracing with dynamic threshold/window adjustments and self-adaptive filters are usually regarded as non-ML, although the related parameters are automatically adjusted according to the input in real time. In this Guide, algorithms with distinguishable training and testing processes are classified as ML-based ones. For algorithms with an implicit learning process, conventions in the field are followed without making such a strict, nuanced distinction between non-ML and ML algorithms. In addition, the discussion proceeds by observing the functions of algorithms categorized by the aforementioned four steps.

It is worth noting that the algorithms reviewed here are those targeting pulse-like signals from nanopore sensors. They are not meant for treating the DNA/RNA/protein sequencing data that may also come from a nanopore sequencer. Processing such sequencing data belongs to a different field in bioinformatics. However, pulse-like signals may also be generated in other sensor devices such as nanogaps^23,24^ and ion channels,^25^ which will be briefly covered here when appropriate. Furthermore, “model” is used in this Guide to exclusively refer to physical models not algorithms. It is important to note that “model” is also widely used in the area of signal processing to represent a realization/implementation of algorithms, especially for ML algorithms.

## Non-ML-Based Signal Processing for Nanopore Sensing

### Step 1. Denoising

**Figure 3:**
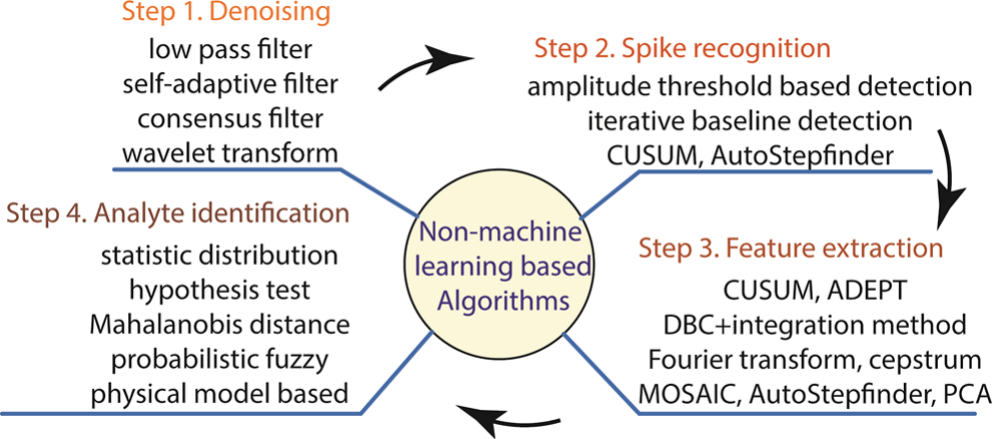
Architecture of non-ML-based algorithms with representative approaches used for each signal processing step.

Traditional methods of signal processing usually rely on low-pass filters for denoising as the first step in Figure 3. It should be emphasized that low-pass filtering is a must for signal amplification and data acquisition in a hardware system to define the bandwidth, mitigate out-of-band noise, and achieve anti-aliasing before digitalization. In this guide, the discussed low-pass filter refers, instead, to the software realization as a category of algorithm for already acquired digital data during the signal processing. In a nanopore system, the current noise power spectrum density, *S*_I_, consists of several different components in distinct frequency ranges.^26,27^ A white thermal noise exists at all frequencies in the spectrum with its power density being inversely proportional to the electrical resistance of the nanopore. The low-frequency noise at frequencies below 1 kHz is usually contributed by flicker noise originating from the charge fluctuation on the pore wall and/or number fluctuation of ions in the pore and 1/*f-shape* noise from electrodes.^26^ In the high-frequency range beyond 1 kHz, the noise power is dominated by the dielectric noise and capacitance noise. The former comes from the dielectric loss of the nanopore membrane, while the latter is a result of current fluctuation generated by the voltage noise of the amplifier input port on the impedance of the nanopore. Considering the frequency distribution of noise power, *S*_I_Δ*f*, the high-frequency range dominates. Therefore, low-pass filters can efficiently restrict the bandwidth of the signal and filter out background noise.^28^ However, the limited bandwidth degrades the capability of capturing fast translocation events and mars important details for analyzing the translocation waveform.

Traditional low-pass filters set a hard frequency threshold for the system. During noise filtration, it may also filter out the high-frequency components of the signal that may contain abundant details about the analytes. Therefore, different approaches have been sought to bypass this dilemma. Backed by the estimation theory, a Kalman filter has been developed to denoise the nanopore sensing signal.^8^ Key parameters of the Kalman filter are adjusted dynamically according to the historical inputs. The stochastic properties of the signal are acquired and represented by these dynamic parameters. Thus, the Kalman filter is capable of extracting a signal whose frequency spectrum overlaps with that of the background noise. In addition, a filtering technology based on wavelet transform has been involved for nanopore signal denoising.^29,30^ First, a group of proper bases that trades between the resolution of time and frequency is selected. Second, the wavelet transform of the input signal is implemented on these bases. Signal and background noise become separable in the wavelet domain even if they overlap in the frequency domain. Finally, a few large-magnitude wavelet components are kept, while the rest of the small-magnitude components are regarded as noise and meant to be removed, because with the specific bases chosen, those large magnitude components are the outcome of the wavelet transform of the main features (information) of the signal. The boundary between large and small magnitude is carefully selected by implementing different threshold functions that work similarly as the cutoff frequency of a traditional low-pass filter. The separability can be further enhanced by adopting multiple levels of wavelet transform, and a simple achievement for discrete signals is a bank of low- and high-pass filters.^30^ By confirming the consistency of signals from multiple readout channels of the same nanopore, a consensus filter is adopted to remove the uncorrelated events as noise from each channel.^31^ In addition, a weighted network among the single nodes gradually builds up and converges to stable values of the weights. This network can deliver consentient events, i.e., the highly correlated signals from each node, and abandon the uncorrelated events, i.e., noise.

### Step 2. Spike Recognition

Spike recognition usually begins with defining an amplitude threshold as a criterion to separate spikes from noise. Apparently, this threshold plays a decisive role in further processing.^32−34^ If the amplitude of a spike surpasses this threshold with reference to the baseline, it is recognized as a translocation event. Otherwise, it is regarded as noise. The identified spike segments are singled out from the current trace, and the associated features are extracted in the next step. Setting a large threshold increases the risk of omitting translocation spikes, misleadingly rendering a low translocation frequency. On the other hand, having a small threshold can mistakenly lead to assignment of noise fluctuations as translocation spikes, thereby incorrectly increasing the translocation counts.^35^ To reduce the subjectivity due to involvement of the user in the threshold selection, the background noise level can be used as a reference.^5−10^ As an example, a certain multiple of the root-mean-square (RMS) value of the background noise can be taken as the threshold.^34,36^ Nonetheless, two potential subjectivity risks persist. The determination of the multiple of the noise level is usually based on the user’s empirical experience. An accurate measurement of the background noise level, e.g., RMS value or peak-to-peak value, is related to the baseline detection. It is common for an algorithm for dynamic baseline detection to be designed to track the baseline position as an effort to mitigate the influence of shift, drift, and slow swing of the baseline on spike recognition. A dynamic average with a proper window size is a simple and straightforward method to obtain the baseline.^32^ How to optimize the window size is crucial for the final performance. A large window can function as a low cutoff frequency filter and shows a stable baseline, but it can be insensitive to rapidly changing signals, including sudden jumps from the baseline. With a small window, changes from the baseline can be followed better, but the penalty is that the attained baseline can be easily influenced by translocation spikes. A simple fixation to overcome this dilemma is to keep the baseline level not updated during the blockage state, i.e., within a spike.^33^ An iterative detection method is further proposed^34^ wherein the baseline is first traced using a simple dynamic average method. Then, the translocation spikes are identified with respect to the baseline. They are then removed from the signal. By repeating the described operations several times, more spikes are recognized and subsequently removed. The dynamic average baseline eventually approaches the real level.

An alternative to the window size selection is to closely follow the current changes without differentiating them in the open-pore state (baseline) from those in the blockage state (spike). A dedicated algorithm named Cumulative Sums (CUSUM) has been developed along this line.^9^ By adopting an adaptive threshold, which is dynamically adjustable according to the slow fluctuations of the signal level, it can detect abrupt changes possibly associated with state switching, e.g., from open-pore to blockage, from a shallow blockage level to a deep level, etc. First, an initial value of the signal level is set by referencing to the average of a small section of the signal at its start. Second, the deviation between the predicted signal level and the real value is calculated and accumulated. If the predicted level is close to the real one, the noise fluctuations above and below this level cancel each other. If the current jumps to a different level, a net deviation accumulates. Third, once this deviation surpasses a user-defined threshold, an abrupt change is identified, and the predicted level is shifted to the new level. Otherwise, the predicted level is updated by averaging the present data points. This algorithm can not only recognize the translocation spikes, i.e., the blockage stage, but also separate multiple levels in one blockage event.^37^ Furthermore, information about these spikes can be extracted naturally, including the amplitude, duration (dwell time in blockage state), interval between adjacent spikes (dwell time in open-pore state), and ionic current levels and dwell time at the corresponding levels for multilevel signals.

### Step 3. Feature Extraction

Once the spikes are singled out from the baseline, feature extraction constitutes the third step in Figure 2. The main features of a spike-like signal commonly include amplitude and duration (width of the spike). An additional parameter to quantify the translocation is the apparent frequency of translocation events (FTE). In general, larger analytes translocating smaller pores induce more severe blockades in the form of deeper spikes; longer analytes with lower translocation speed, caused by weaker driving forces and/or stronger analyte–nanopore interactions, yield longer durations; higher analyte concentrations and/or larger bias voltages give rise to higher FTEs. These are intrinsic factors relevant to the properties of analytes and nanopores. Extrinsically, the bandwidth constraint of an electrical readout system may distort narrow spikes, rendering an attenuation of amplitude and a prolongation of duration. Signal distortion by limited bandwidth has received quantitative analysis.^28,38^ In order to recover the true spike waveform from the distorted one, a physical model-based algorithm named ADEPT^39,40^ and a Second-Order-Differential-Based Calibration (DBC) method with an integration method^41−43^ have been developed. The ADEPT algorithm is based on an equivalent circuit model of the nanopore system. From the system transfer function of the circuit, the true signal is recovered from the distorted one by inversely applying the system function. Thus, the affected spikes corresponding to short-lived events are compensated for to restore the unaffected features. In the DBC method, a Fourier series is first applied to fit the translocation spikes for smooth waveforms. Second, the second-order derivative of the smoothed waveform is calculated. Third, the minima of the derivative are located at positions corresponding to the start and end time points of the translocation, thereby leading to an accurate determination of the duration of a spike. Finally, the attenuation of amplitude by the limited bandwidth is compensated for by considering the area beneath the spikes referred to as the baseline. The DBC algorithm has been integrated in software packages for signal processing of nanopore sensing data.^33,44^

ADEPT is effective for short-duration spikes, while CUSUM is suitable for long-duration spikes with multiple blockage levels. A software platform, MOSAIC, has emerged by combining the two algorithms to benefit from their respective strengths.^32^ An advanced version of CUSUM has recently been adopted in MOSAIC for a robust statistical analysis of translocation spikes. In addition, an algorithm named *AutoStepfinder* is devoted to stepwise signals.^45^ First, the initial number of step levels representing different blockage states is assigned. Fitting is then implemented to achieve the minimum error. Second, the fitting outcome is evaluated and compared with the halt condition for the required accuracy. Third, if it does not reach the halt condition, the number of step levels is gradually increased for the new iterative round of data fitting. In an iterative manner, this process is repeated until finding the best number of step-levels. This algorithm is developed for signals arising from the growth dynamics of protein polymer microtubules with optical tweezers.^46^ Translocation spikes from nanopore sensors, including the typical single-step signals and blockages with multiple levels, are all targets of this algorithm. Other multiple-step signals from electrical, optical, and mechanical measurements can also be processed using this algorithm.^10,45^ For stepwise signals, the Rissanen principle of Minimum Description Length (MDL) is adopted to identify the steps, e.g., the close–open dynamics of ion channels.^47^ Here, an anticipated location of step is confirmed by achieving an MDL, which trades off between fineness and fitting accuracy.

Besides the three main features of spikes, amplitude, duration, and FTE, more specific features need to be scrutinized for analyte classification and analysis. A large number of different features, i.e., multiple dimensions of feature space, advances the especially powerful ML-based classifiers for processing data in high-dimensional space. In contrast, simple non-ML-based classifiers, such as statistical distribution-based distances and hypothetical tests, provide limited functionalities. The ML-based classifiers will be discussed later, and the focus is placed on feature extraction here. Several details of the translocation spikes are selected as features, such as increasing and decreasing slopes of a spike, spike area, area of increase and decrease period and their ratio, symmetry of spikes, bluntness of spikes, and “inertia” with respect to the current axis and time axis with and without normalization by the amplitude.^48−50^ The frequency *spectrum* and *cepstrum* of spikes based on the Fourier transform can also be used as features for the classification.^51^ Usually, peaks may appear in the frequency spectrum representing the major frequency components of a signal. The features of these peaks, e.g., the position, amplitude, and phase angle of the peaks, are collected as features of the signal in the frequency domain. Furthermore, if no clear peak-wise pattern appears, the amplitude and phase angle of a series of frequency points obtained by resampling on the spectrum can be used as features for classification as well.^51^ If the number of features is too large and some of them are highly correlated, the Principal Components Analysis (PCA) method can be employed to compress the redundant information and decrease the dimensionality of the feature space. This treatment can lead to refined features in an efficient manner for the classification algorithms.

### Step 4. Analyte Identification

The final step of signal processing is to infer the analyte properties and identify/classify the analytes based on the extracted features. As discussed above, simple physical models can be utilized to correlate the amplitude of spikes to the size and shape of analytes;^52,53^ to relate the duration to the translocation speed and nanopore–analyte interaction that in turn are connected to the physiochemical properties such as mass, charge, dipole, and hydrophobicity;^54,55^ and to associate the frequency of spikes to the concentration of analytes at a given bias voltage.^55,56^ By synthetically considering the three features in a three-dimensional space and utilizing the tools of hypothetical tests, the separability of spike clusters in a feature space can be inferred, each cluster can be attributed to certain characteristics of the analytes, and new spikes can be identified to one of these clusters. For example, the Mahalanobis distance metrics are adopted to assess the similarity of certain spikes with labeled clusters in the feature space so that five different amino acids can be identified.^57^ Moreover, a probabilistic fuzzy algorithm is adopted to quantify the concentration range of analytes through a comparison between the Gaussian distribution of the blockage amplitude and the calibration values.^58^ The fuzzy property endows the algorithm with flexibility, which can tolerate the data variation from the experimental conditions to some extent. Details of translocation waveform are considered by invoking more sophisticated physical models^55,59^ to distinguish proteins based on their fingerprint feature of blockage of current distribution.^60^

Instead of DC bias, an AC voltage can be applied as excitation and the corresponding AC current is recorded as signal.^61^ It has been shown that the frequency response properties, including magnitude and phase, of translocating nanoparticles, SiO_2_, Au, and Ag, are easily differentiable by employing this AC method.

The non-ML-based algorithms for processing nanopore signals are summarized in Table 1, while the commonly used algorithms in each step are depicted in Figure 3.

**Table 1:** Summary of Non-ML-Based Algorithms^a^

name	function	processing flow step	input	output	comments	refs
Low-pass filter	Denoise	1	Raw TSD	Clean TSD	Simple and commonly used for pretreatment of raw data	(26),^28^
Kalman filter	Denoise	1	Raw TSD	Clean TSD	Self-adaptive filtering based on parameter estimation theory	(8)
Wavelet transform filter	Denoise	1	Raw TSD	Clean TSD	Filtering in wavelet domain	(29),^30^
Consensus filter	Denoise	1	Raw TSD	Clean TSD	A network excluding incoherent component of data among different nodes as noise	(31)
Amplitude threshold-based spike recognition	Identify spikes	2	Raw/clean TSD	SS	Easy and commonly used; A user-defined threshold needed	(32−36)
Dynamic average method	Trace baseline	2	Raw/clean TSD	Baseline of TSD	Easy and commonly used	(32),^33^
Iterative baseline detection	Trace the baseline	2	Raw/clean TSD	Baseline of TSD	Efficiently excluding the influence of spikes during baseline tracing	(34)
Cumulative Sums (CUSUM)	Trace the current change and extract SF	2, 3	Raw/clean TSD	SS and SF: amplitude, start and end time	Accumulated deviation between the true trace and predicted level used as an indicator for current level changes	(9),^37^
ADEPT	Extract SF	3	SS	SF: amplitude, duration	Physical model based; Recovery of unaffected spike waveform from distortion caused by bandwidth limitation of the signal readout circuit	(39),^40^
Second-Order-Differential-Based Calibration (DBC)	Extract SF	3	SS	SF: amplitude, duration	Accurately locating the start and end time of translocation spikes by minima of second-order derivative of the spike waveform	(41−43)
Modular single-molecular analysis interface (MOSAIC)	Extract SF	2, 3	Raw/clean TSD	SF: amplitude, duration	An integrated software combining ADEPT and CUSUM+ for short and long events, respectively	(43)
AutoStepfinder	Extract steps in signal	2, 3	Raw/clean TSD	SF: step height, duration	Iterative way to find the number of step levels resulting in the best fit	(45)
Rissanen’s minimum description length (MDL)	Extract steps in signal	2, 3	Raw/clean TSD	SF: step height, duration	Minimizing description length of data segment to locate steps in signal; a trade-off between fineness and fitting accuracy	(47)
Principal Components Analysis (PCA)	Compress feature space	3	SF	Compressed SF	Reducing redundant information and decreasing feature space dimension by canceling the linear dependency among feature variables	(52)
Mahalanobis distance metrices	Classify analytes	4	SF	Class label	Measuring the Mahalanobis distance of points in feature space to judge the similarity	(57)
Probabilistic fuzzy algorithm	Classify analytes	4	SF	Class label	Clustering data in feature space by consideration of their statistical distribution in a probabilistic way	(58)
Rotational dynamic current fluctuation model	Extract analyte properties	3, 4	SS	Properties of proteins	Extracting five parameters of proteins: shape, volume, charge, rotational diffusion coefficient, and dipole moment	(60)

a TSD: time sequence data, i.e., the ionic current trace in nanopore sensors; SS: spike segment, i.e., the current trace segment of a translocation spike; SF: spike features.

## ML-Based Signal Processing for Nanopore Sensing

The ML-based algorithms are mainly devoted to treating two key aspects in a typical signal processing flow, i.e., spike recognition in step 2 and analyte identification in step 4. Besides, few algorithms are developed for step 1, denoising, and step 3, feature extraction.

### Step 1. Denoising

A Deep Neural Network (DNN) can be adopted to filter out the noise from the signals generated by carboxylated polystyrene nanoparticles translocating a 5-μm-long nanochannel.^62^ In such an algorithm, the time sequence traces as signals are first sent to a convolutional autoencoding Neural Network (NN) that repeats the convolution of input and passes on the features to the next stage, i.e., an activation function of either rectified linear unit (ReLU) or LeakyReLU. This operation converts current traces into vectors in a high-dimensional feature-enhanced space by keeping the features and dropping the time resolution. Next, the vectors undergo deconvolution to reconstruct the current trace in the original size. During the training process, the weights and biases for each node in the NN are tuned by means of gradient descent optimization to evaluate the deviation between the output and the denoised (control) current traces obtained by Fourier analysis and wavelet transform. In this way, the algorithm can automatically identify features and discard noise in the high-dimensional feature space, thereby overcoming the limitation of traditional filtering with overlapping frequency components of signal and noise. This is a typical unsupervised algorithm needing no labeled data sets, i.e., the ideal “clean” data without noise, during the training process.

### Step 2. Spike Recognition

Regarding Step 2, most efforts are based on the Hidden Markov Model (HMM) strategy.^63,64^ The HMM is naturally suitable for the description of stochastic hops between the open-pore state and the blockage state, as a Markov chain. The key to train an HMM is to determine the probability of state transition from one to the other, i.e., state transition probability, and the probability of ascertaining the value of an observed variable with certain values of hidden stochastic variables, i.e., output probability. In order to train the HMM, labeled data sets are necessary. For nanopore translocation signals, the current of each sampling point need be assigned to a given state, e.g., open-pore, shallow blockage, deep blockage, etc. First, a Fuzzy c-Means (FCM) algorithm and a Density-Based Spatial Clustering of Applications with Noise (DBSCAN) have been adopted to cluster the sampled data. Next, the Viterbi approach, which is used to obtain the maximum *a posteriori* probability and estimate the most likely sequence of hidden states in an HMM, has been utilized to achieve an intelligent retrieval of multilevel current signatures. This approach has enabled detection of nanopore translocation events and extraction of useful information about single molecules under analysis.^65,66^ Lately, some feature vectors from HMMs have also been used to provide the characteristics of translocation spikes.^64,67^ Finally, the feature vectors are used for further analyte classification. The components of feature vectors include not only translocation spike related features, such as mean value and variation of spike amplitude, but also stochastic process related parameters, such as the transition probability between the open and blockage states and the statistical distribution of emission probability.

Concerning classification and by way of introduction to such a topic, HMMs have also been utilized for classification. For instance, HMM-based duration learning experiments on artificial two-level Gaussian blockade signals can be used to identify a proper modeling framework.^68^ Then, the framework is applied to the real multilevel DNA blockage signal. Based on learned HMMs, base-pair hairpin DNA can be classified with an accuracy up to 99.5% on experimental signals.

### Step 3. Feature Extraction

As to Step 3, most commonly used algorithms are non-ML-based. Few studies on ML algorithms can be found though. Based on the Residual Neural Network (ResNet), a bipath NN, named Bi-path Network (B-Net), has recently been established to extract spike features.^35^ Since the task of counting the number of spikes is essentially different from that of measuring the amplitude and duration, the bipath design, composed of two ResNets, each one trained for one task, has been shown to be robust with compelling performance. During the training process, segments of time sequence traces are first sent to the NN. The predicted values of spike number, average amplitude, and duration of the appearing spikes are then compared with the respective ground truths. Next, the deviations of the predicted values and the ground truths are back-propagated through the NN using the Stochastic Gradient Descent (SGD) algorithm and the weights of each node are adjusted. Finally, the training performance in each epoch is evaluated on a validation data set so that the best trained NN is selected. The training data sets are artificially generated by a simulator on the foundation of a set of physical models, describing open-pore current, blockage spikes, background noise, and baseline variations. The trained B-Net can directly extract the three features of spikes, i.e., amplitude, duration, and number (or FTE), from raw translocation data of λ-DNA and protein streptavidin. The features show clear trends with the variation of certain conditions, which is in agreement with the corresponding physical mechanisms of analyte translocation. The B-Net avoids the inherent subjectivity found on spike recognition with traditional threshold-based algorithms that are dependent on a user-defined amplitude threshold.

A new concept of shapelet has been involved in the feature extraction of translocation spikes.^69^ Shapelets are short time-series segments with special patterns that contain discriminative features. For translocation spikes from nanopore sensors, the tiny fluctuations of the ionic current in the blockage state, i.e., the bottom of the blockage spike, do not always result from noise. They can be characterized by certain regular patterns representing the characteristics of the translocating analytes as well as their interactions with the nanopore. In the learning time-series shapelets (LTS) algorithm, these regular patterns are learned as shapelets automatically from the training data set to maximize the discriminative features among the spikes from different analytes. Then, the similarities of test spikes and these shapelets are measured by the Euclidean distance, as the features of these spikes. Consequently, a multidimensional feature space is established. On the platform of aerolysin nanopore, the LTS algorithm is proven to have the ability to discriminate the translocation spikes generated by 4-nucleotide DNA oligomers with single-nucleotide difference.^69^

### Step 4. Analyte Identification

Finally, when it comes to Step 4, main trends have been directed toward three approaches, (i) Support Vector Machines (SVMs), (ii) Decision Trees (DTs) and Random Forests (RFs), and (iii) NN-based classifiers. For shallow ML algorithms such as (i) and (ii), the inputs are the features of a signal, i.e., vectors in high-dimensional feature space. The extraction of features, i.e., construction of feature space, are adequately discussed in Step 3 of non-ML-based signal processing algorithms as well as in the previous section. The commonly used features include those from the time-domain of signal, e.g., amplitude, duration, and frequency of spikes, and those from frequency-domain, e.g., peaks in the spectrum. Regarding deep-learning (DL) algorithms such as (iii), the inputs are usually the time sequence of current traces or spike segments. Therefore, the DL algorithms, compared to their shallow ML counterparts, may avoid the tedious feature extraction process that usually needs rich experience and can be subjective. An expanded discussion of this issue is found in the section Strategies of ML-Based Algorithms. However, although rare, it is also found that extracted features have been used as inputs for DL algorithms.^70^

An SVM is a linear classifier whose goal is to find a hyperplane in an *n*-dimensional space that segregates data points belonging to different classes. Consequently, data points falling on either side of the hyperplane can be attributed to different classes. Multiple possible hyperplanes can be chosen to separate two classes of data points. The main aim of an SVM is to find the plane with the maximum margin, i.e., the maximum distance between the data points of both classes. Maximizing the margin distance provides robustness such that future data points can be classified with high confidence. Support vectors are the data points closer to the hyperplane, which are taken as references and determine the position and orientation of the hyperplane, thereby maximizing the margin of the classifier. Since an SVM is a linear classifier, it works better when there is a clear margin of separation between classes, and it is more effective in higher dimensional spaces, i.e., when the number of dimensions is greater than the number of samples. This algorithm does not incorporate nonlinearities to the input points by itself. However, complementary kernels can be involved to realize the nonlinearity. The cost is that they come with the incorporation of more dimensions to the inputs and carry more processing loads. Accordingly, the SVM does not perform well when the data points at different target classes severely overlap. As a result, this algorithm needs a preprocessed data set in its input to build up the high-dimensional feature space. Such a preprocessing step is not linked to the automatic optimization process of the algorithm and has to be conducted using human-engineered tools, which makes this procedure less automatic and adds limitations derived from human subjectivity.^71^

In regard to how to utilize SVMs to attain classification, a strategy has been introduced to classify and interpret nanopore and ion-channel signals.^72^ The Discrete Wavelet Transform (DWT) is used for denoising nanopore signals and features. Spike duration, amplitude, and mean baseline current are extracted and subsequently used to detect the passage of analytes through the nanopore. First, a two-stage feature extraction scheme adopts the Walsh-Hadamard Transform (WHT) to provide feature vectors and PCA to compress the dimensionality of the feature space. Afterward, classification is carried out using SVMs with 96% accuracy to discriminate two highly similar analytes. Along the same lines,^73^ each current blockade event can be characterized by the relative intensity, duration, surface area, and both the right and left slope of the pulses. The different parameters characterizing the events are defined as features and the type of DNA sample as the target. By applying SVMs to discriminate each sample, an accuracy between 50% and 72% is shown by using two features that distinctly classify the data points. Finally, an increased accuracy up to 82% can be achieved when five features are implemented. Likewise, the SVM has also been used to identify two different kinds of glycosaminoglycans with an accuracy higher than 90%.^74^

Similarly to nanopore techniques, nanogap sensors generate characteristic tunneling current spikes when individual analytes are trapped in a gap between two electrodes. As is the case for nanopores, this technique has also been used to identify individual nucleotides, amino acids, and peptides at a single-molecule level. Following this line of research using nanogaps, an SVM has been shown to classify a variety of anomers and epimers via the current fluctuations they produce when being captured in a tunnel junction functionalized by recognition probe molecules.^24^ Likewise, a tunneling nanogap technique to identify individual RNA nucleotides has been demonstrated.^51^ To call the individual RNA nucleotides from the nanogap signals, an SVM is adopted for data analysis. The individual RNA nucleotides are distinguished from their DNA counterparts with reasonably high accuracy. In addition, it is found through using an SVM for data analysis how probe molecules in a nanogap sensor distinguish among naturally occurring DNA nucleotides with great accuracy.^75^ It is further shown that single amino acids could be identified by trapping the molecules in a nanogap being coated with a layer of recognition molecule probes and then by measuring the tunneling current across the junction.^23^ Since a given molecule can bind in different manners in the junction, an SVM algorithm is useful in distinguishing among the sets of electronic “fingerprints” associated with each binding motif.

To pursue classification, ensemble learning is involved in signal processing of nanopore sensing. By assembling the results from multiple simple learners, the ensemble learner can achieve a better generalization than by the individual simple learners.^76^ A simple learner is usually based on learning algorithms with low complexity, such as DT and NN. An ensemble learner combines multiple simple learners based on the same algorithm, i.e., homogeneous ensemble, or different algorithms, i.e., heterogeneous ensemble. To highlight the advantage of assembling, the individual learners should behave differently, yet with sufficient accuracy. Therefore, an important issue in this scheme is to find a smart way to divide the training data sets for the individual learners, especially for homogeneous ensembles, since the behavior of each learner is based on the training data. According to the strategies adopted to generate these base/component learners, ensemble learning algorithms can be divided into two major categories: (i) the individual learners are generated sequentially with strong correlations in between, and (ii) they are generated in parallel with weak correlation.

Boosting algorithms belong to the first category, such as Adaptive Boosting (AdaBoost), in which the training data for each individual learner is selected/sampled from the entire training data set. The performance of the current learner determines the manner in selecting the training data for the next learner, and as mentioned, the simple learners in boosting algorithms are generated one by one. AdaBoost, assembled by multiple DT classifiers, is used to classify the spikes generated by the mixture of two kinds of 4-nucleotide DNA oligomers with single-nucleotide difference.^67^

Bagging, on the other hand, is a typical algorithm of the second category, in which the training data for each learner is selected simultaneously in the training data set. Thus, the learners can be trained individually at the same time. An RF algorithm constructs the bagging ensemble architecture by involving a random selection mechanism in the training data selection for each DT. Thus, the RF shows better robustness and generalization ability than achievable with the simple DT. Moreover, the performances of RF and SVM are contrasted.^77^ On one hand, an SVM-based regressor is used to establish the correspondence between specific peptide features inside the pore and the generated signal. On the other hand, an alternative approach for supervised learning can be explored by implementing the RF regression for translocation waveform prediction. The resulting RF becomes more robust to outliers also exhibiting less overfitting.

To boost the generalization ability, Rotation Forest (RoF) has been proposed. It builds classifier ensembles using independently trained DTs. The RoF is proven to be more accurate than bagging, AdaBoost, and RF ensembles across a collection of benchmark data sets.^78^ In an RoF algorithm, the feature set is randomly split into a number of subsets in order to create the training data for the base classifiers, i.e., DT, and the selected training data for each DT is further processed usually by PCA. Then, a rotation operation is applied in the feature space to form the new features for the base classifiers. The aim of the rotation operation is to boost individual accuracy and diversity simultaneously within the ensemble.

Along the same research line, RoF ensembles have been used to demonstrate label-free single-cell identification of clinically important pathogenic bacteria via signal pattern classification in a high-dimensional feature space.^79^ A similar classifier is used in bacterial shape discrimination^48^ and for label-free electrical diagnostics of influenza to distinguish among individual viruses by their distinct features from the same group.^14,50^ Recently, RoF and RF-based classifiers have been developed to identify four kinds of coronal viruses according to the features of translocation spikes, even when they have highly similar size and shape.^80^ A comparison between RFs and Convolutional Neural Networks (CNNs) has recently been conducted.^81^ Using either a set of engineered signal features as input to an RF classifier or the raw ionic current signals directly into a CNN, both algorithms are found to achieve similar classification accuracy ranging from 80% to 90%, depending on the hyperparameters and data sets.

Another major category of classifiers is those based on DNNs with various architectures, such as CNN, fully connected DNN, Long Short-Term Memory (LSTM), ResNet, etc. The DNNs came to the scene to eliminate an important bottleneck in the previously traditional ML pipeline. Essentially, previous ML workflows put feature extractions from raw data in the hands of human experts in step 3 as discussed above. The consideration behind relying on human expertise is not guided by the optimization conducted in the classifiers whatsoever, in order to attain better discrimination. Great risks are involved in the fact that human judgments could neglect important information and features present in the raw input data. The combinatorial nature of possible correlations among different features cannot be completely contemplated by human expertise. Therefore, essential correlations can be accidentally discarded with the consequential compromises in classification accuracy. Likewise, some feature correlations can be essential in the perspective of human reasoning. Yet, those could also be completely useless statistical features at the time of attaining better classification performance. The DNNs, instead, promote the extraction of features using optimization mechanisms guided by ultimate classification objectives in an end-to-end fashion. By back-propagating errors and applying optimization steps, such as SGD, these architectures modify internal parameters in the networks in order to accomplish better classification performance. Every stage in such multilayer pipelines abstracts more and more relevant information regarding the final objectives of the complete system. The automation of the feature extraction stages in the ML pipeline bypasses an explicit operation in step 3 in Figure 2. Hence, the DNNs can directly process the traces/segments of translocation spikes from step 1 or 2 and achieve outstanding results in a variety of important applications, such as computer vision, speech recognition, and Natural Language Processing (NLP) among many other newer and essential fields.^82^

Following the research line of CNNs, a CNN is developed for a fully automated extraction of information from the time-series signals obtained by nanopore sensors.^83^ It is trained to classify translocation events with greater accuracy than previously possible, which increases the number of analyzable events by a factor of 5.^83^ An illustration of the step-by-step guide in how a CNN can be used for base classification in DNA sequencing applications is available in the literature.^10^ Moreover, a CNN has been adopted to classify different kinds of proteins according to the fluorescently labeled signals from optical measurement of the translocation through solid-state nanopores, which also show spike-like features as electrical current signals.^84^ A comparison with the SVM as a more conventional ML method is provided for discussion of the strengths and weaknesses of the approaches. It is claimed that a “deep” NN has many facets of a black box, which has important implications in how they look at and interpret data. Moreover, each translocation event is described by various features in order to enhance classification efficiency of nucleotide identities.^70^ By training on lower dimensional data and comparing different strategies, such as fully connected DNN, CNN, and LSTM, a high accuracy up to 94% on average is reached. In addition, a ResNet is trained and acquired the ability to classify the spikes generated by the translocation of two kinds of DNA oligomers, 5′–(dA)7(dC)7–3′ and 5′–(dC)7(dA)7–3′ that only differ in the sequence direction.^85^ Prior to classification, an ensemble of empirical mode decomposition, variational mode decomposition, inherent time scale decomposition, and Hilbert transform has been designed to extract multispectral features of nanopore electrical signals. By combining ResNet with SVM, adeno-associated viruses carrying different genetic cargos are discriminated according to their respective translocation spike signal through a SiN_*x*_ nanopore.^86^ The ResNet extracts abstract “features” of the signal traces, although these features are not describable and cannot be directly correlated to physical meanings, and delivers them to a SVM for classification.

Besides the translocation spike signals, a DL algorithm based on CNNs and LSTM architecture can also be used for recognition of the open and blocked states of ion channels by the ionic current levels.^25^ It can process signals from multiple channels with multiple ionic current levels. The algorithm is completely unsupervised and, thus, adds objectivity to ion channel data analyses.

An NN-based technique called Positive Unlabeled Classification (PUC) has been introduced to learn the interference of background noise fluctuation with the spikes generated by the four different nucleotides in a nanogap sensor. It uses groups of training current signals recorded with the target molecules. By combining with a common NN classifier, it can identify the four nucleotides with a high degree of precision in a mixture sample.^87^

Other ML optimization algorithms such as Expectation Maximization (EM) have also been used for classification. An EM is a widely used iterative algorithm to estimate the latent variables from the observations of the statistical estimation theory. The unobserved latent variables can be induced from the incomplete/damaged data set or variables that cannot be measured/observed directly. In an EM algorithm, the two steps, E and M, are repeated alternatively. In the E step, the values of the latent variables are estimated from the parameters of the stochastic schemes. In the following M step, the stochastic parameters are updated according to the observed variables from the data set and latent variables from the previous E step. By iterating these two steps, the stochastic parameters may converge to their real values. The EM algorithm is adopted in several clustering methods, such as the k-mean clustering and the Gaussian mixture model. The EM algorithm has been implemented to cluster the translocation spikes from wild-type *E. coli* cells and *fliC* deletion mutants.^88^ Seven features related to the shape of the translocation spikes are selected, and the statistical distribution parameters of the spikes in a seven-dimensional feature space are estimated by applying the EM iteration. In addition, the same algorithm has been used to classify two viruses, influenza A and B, through the translocation signals from peptide functionalized SiN_*x*_ nanopore sensors.^49^ Other classification and clustering algorithms have also been implemented to identify various analytes via the translocation features obtained from nanopore sensors, such as the *k*-nearest neighbor (*k*),^89,90^ the logistic regression,^69,89,90^ and the naive Bayes.^89^

An important facet of the ML related algorithms is that they devote significant effort to comparisons among as many methods as possible. For instance, by utilizing ML, it is possible to determine two different compositions of four synthetic biopolymers using as few as 500 events.^89^ Seven ML algorithms are compared: (i) AdaBoost; (ii) *k*-NN; (iii) naive Bayes; (iv) NN; (v) RF; (vi) logistic regression; and (vii) SVM. A minimal representation of the nanopore data, using only signal amplitude and duration, can reveal, by eye and image recognition algorithms, clear differences among the signals generated by the four glycosaminoglycans. Extensive molecular dynamics simulations and ML techniques are used to *featurize and cluster* the ionic current and residence time of the 20 amino acids and identify the fingerprints of the signals.^90^ Prediction is compared among three classifiers: *k*-NN with *k* = 3, Logistic regression, and RF with the number of estimators of 9.^90^

**Figure 4:**
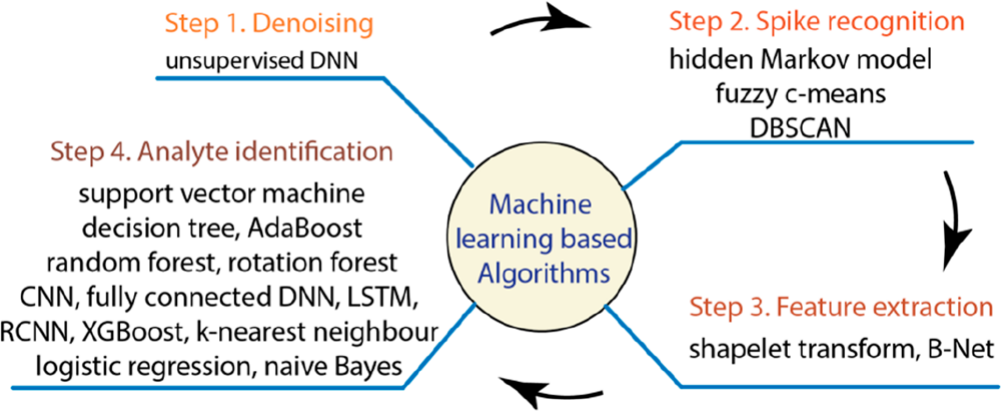
Architecture of ML-based algorithms with representative approaches used for each signal processing step.

The ML-based algorithms for processing the nanopore signals are summarized in Table 2, while the commonly used algorithms in each step are depicted in Figure 4.

**Table 2:** Summary of ML-Based Algorithms^a^

name	function	processing flow step	input	output	comments	ref
U-Net	Denoise	1	Raw TSD	Clean TSD	Based on deep learning neural network; involving convolution and deconvolution processes; An unsupervised learning	(62)
Hidden Markov Model (HMM)	Trace the current change and Recognize spikes	2	Raw/clean TSD	SS	Probabilistic graphical model	(64−66,68)
Fuzzy-c means (FCM)	Cluster current to different levels	2	Raw/clean TSD	Current with state/level label	Clustering algorithm used for labeling current data for HMM	(66)
Density-based spatial clustering of applications with noise (DBSCAN)	Cluster current to different levels	2	Raw/clean TSD	Current with state/level label	Clustering algorithm used for labeling current data for HMM; No need to define number of states/levels	(65)
Deep-channel	Cluster current to different levels	2	Raw/clean TSD	Current with state/level label	Combination of CNN and LSTM; unsupervised learning	(25)
Bipath neural network (B-Net)	Extract SF	2, 3	Raw/clean TSD	SF: amplitude, duration, frequency	Use of two ResNet branches, essentially different tasks for different branches	(35)
Learning time-series shapelets (LTS)	Extract SF	3	SS	SF: patterns in the blockage waveform	Automatic recognition and summarization of patterns in time domain translocation waveform	(69)
Support Vector Machines (SVMs)	Classify analytes	4	SF	Class label	Support vectors in feature space separating data from different classes	(23, 24, 51, 72−75)
Decision tree (DT)	Classify analytes	4	SF	Class label	Structuring features and judging probabilistically	
Random forest (RF)	Classify analytes	4	SF	Class label	Ensemble learning composed by DTs	(77),^81^
Rotation forest (RoF)	Classify analytes	4	SF	Class label	Ensemble learning with advanced treatment of data to enhance robustness	(14, 48, 50, 78, 79)
Adaptive boosting (AdaBoost)	Classify analytes	4	SF	Class label	Ensemble learning composed by simple learners, e.g., DTs, NNs, naive Bayes, etc.	(67)
Convolutional neural network (CNN)	Classify analytes	4	SS	Class label	Adaptation from 2D image data to 1D TSD; supervised DL	(10, 25, 70, 83)
Fully connected deep neural network (DNN)	Classify analytes	4	SS	Class label	Supervised DL	(70)
Long–short-term memory (LSTM)	Classify analytes	4	SS	Class label	Mimicking weight adjustment strategy of real neurons; supervised DL	(25, 70)
Residual neural network (ResNet)	Classify analytes	4	SS	Class label	Skipping connections or shortcuts to jump over some layers; supervised DL	(85)
Stochastic gradient descent (SGD)	Quantify back-propagating errors	4	Current output and correct output	Adjust values for each weight and bias	Back-propagation algorithm for NN based DL	
Positive unlabeled classification (PUC)	Classify analytes	4	SS	Class label	Recognition of noise interference in first step and classification in second step of signal processing; unsupervised learning.	(87)
Expectation maximization (EM)	Classify analytes	4	SF	Class label	Iteration between E step and M step.	(49, 88)
*k*-nearest neighbor (*k*-NN)	Classify analytes	4	SF	Class label	Lazy learning; classification by class of nearest data	(89, 90)
Logistic regression	Classify analytes	4	SF	Class label	Linear regression for logit^b^	(69, 89, 90)
Naive Bayes	Classify analytes	4	SF	Class label	Probabilistic decision to minimize the total Bayes risk	(89)

a TSD: time sequence data, i.e., the ionic current trace in nanopore sensors; SS: spike segment, i.e., the current trace segment of a translocation spike; SF: spike features; NN: neural network; DL: deep learning.

b In ML, Logit is a vector of raw (non-normalized) predictions that a classification algorithm generates, which is usually then passed to a normalization function. Such a normalization function maps the real number line (-inf, inf) to probabilities [0, 1]. If the classifier solves a multiclass classification problem, logits typically become an input to a softmax function (normalization function). The softmax function then generates a vector of (normalized) probabilities with one value for each possible class.

## Strategies of ML-Based Algorithms

As its name alludes, ML conforms to a set of algorithms that improve automatically through experience. Such algorithms are essentially machines that learn a task from data. One part of these algorithms is mainly regarded as classification machines that use preprocessed features as inputs. The conventional ML techniques, *classical MLs*, are limited by the information contained in such features, since they are obtained by other algorithms tailored by highly specialized human engineering. Such specialization is bound by human subjectivity, which does not always align with the best decisions at time of providing relevant features to the classifiers for the task at hand. The typical classical ML algorithms for signal processing of nanopore sensing are *k*-NN, DT, RF, RoF, AdaBoost, and SVM among others.

Following the categorization scheme laid down and the line of argument thus far, all these algorithms share the same strategy of receiving highly preprocessed human engineered features. This approach limits their capability of self-discovering relevant features in order to attain a higher performance for the task. *Deep Learning*, on the other hand, is based on representation learning, which is a set of strategies by which representations of data can be learned automatically to extract useful information when building, for instance, a classifier.^91^

To extract the features, clear criteria in traditional algorithms for preprocessing data need consequently be defined and described by unambiguous logic judgments. These criteria are usually based on users’ empirical experience, thereby rendering them subjective and case dependence. For example, a user needs to summarize related key features of the spikes by observation and experience in order to single out the spikes from a noisy background in step 2, e.g., the threshold for spike recognition. The prevalent algorithms used in these application scenarios follow this path, and all the links in the path should be expressed explicitly. The limitation for each step is obvious; feature extraction needs experts, but some key features may only bear limited information. The criteria are rigid and stiff, which can be incompatible with highly nonlinear cases turning to an even more complicated and sophisticated structure. Such limitations can be attributed to the weakness of the concept itself, since the traditional algorithms request an explicit representation of everything, including features, variables, and logical relationships. This process inexorably invites subjectivity.

With DL algorithms, in contrast, the features of spikes are acquired by the algorithm during the training process and thus include as much of the original information as possible. This approach inherently bears the commonality for wide application scenarios. Its assessment process is flexible and probabilistic, thus implying a more complex and nonlinear logic and indicating a powerful method with robust performance. The whole process minimizes the participation and intervention of users, which warrants a maximum level of objectivity.^91^ The automatic feature extraction in DL algorithms is specially beneficial for atypical signals induced by nanopore–analyte interaction, morphology change dynamics, adsorption–desorption, and clogging. Such atypical signals usually do not display the spike-like features of the typical translocation signals. Therefore, it is challenging and requires rich experience to define and extract features for those signals.

The DL strategy builds its own features by highlighting the most explanatory ones, while diminishing the ones with the least explanatory value for the task that the network is commissioned to solve. Such an efficacy is achieved because the feature extraction part of the network uses optimization mechanisms connected to the final optimization algorithms in the pipeline, which are regarded to address the final task. Such connections are provided by back-propagating errors throughout the architecture in a scheme that utilizes derivatives, the chain rule, and SGD. These mechanisms work together on moving the optimal point of the network progressively in order to find some local minimum in a loss function that the system seeks to minimize. Consequently, the feature extractor mechanism harmoniously follows more robust paths of optimization that permit the whole network to achieve the optimum performance.^91^

Historically, the main differences in the data to be processed are forwarded to the corresponding DL architectures. Thus, Feed-Forward Artificial Neural Networks (FFANNs) process data in a way that information flows from input to output without a loop in the processing pipeline. In other words, the input to any module in the network is not influenced by the outputs of such modules directly or indirectly. Examples of FFANN include CNN implementations, such as LeNet-5 introduced in 1998 and known as CNNs today.^92^ Later, AlexNet was introduced in 2012^93^ with a considerably larger but structurally similar architecture (60,000 parameters for LeNet-5 vs 60 million for AlexNet). Then, VGG-16, developed in 2014, introduced a deeper (with 138 million parameters) yet simpler variant of the previous architectures.^94^ Inception network (or GoogleNet) was also introduced in 2014,^95^ with its 5 million parameters in version V1 and its 23 million in version V3. As networks started to become deeper and deeper, it was noticed that adding more layers would compromise the quality of gradients. The advantage of the network concept could eventually vanish or explode exponentially with the number of layers. Nowadays, this limitation can be mitigated by employing a new architecture called ResNet, which incorporates skip connections to residual layers. There are several ResNet variants, for instance, ResNet 50 with 25 million parameters. Another architecture called ResNeXt is an extension of ResNet by replacing the standard residual block with one having a different strategy.^96^ Finally in the DenseNet architecture, the feature map of each layer is concatenated to the input of every successive layer within a dense block. This strategy encourages feature reuse thus allowing later layers within the network to directly leverage the features from earlier layers. Compared with ResNet, DenseNets are reported to possess better performance with less complexity.^97^ For instance, DenseNet has architectures with the number of parameters ranging from 0.8 million to 40 million.

Concomitantly, Recurrent Neural Networks (RNNs) allow the existence of loops in the pipeline. Derived from FFANNs, RNNs use their internal state (memory) with temporal dynamic behaviors to process variable-length sequences of inputs. Basically, an RNN uses sequential data, i.e., time series, all regarded as temporal problems in language translation, sentiment classification, NLP in general, Automatic Speech Recognition (ASR),^98^ image captioning, music generation, etc. Their memory from prior inputs influences the current network’s internal state and output. An important property of RNNs is that they share weights along the sequence and apply Backpropagation Through Time (BPTT) throughout the sequence in order to learn.^98^ The main principle of BPTT is the same as the traditional back-propagation, where errors are back-propagated from its output layer to its input layer. However, the BPTT differs from the traditional approach in that it sums up errors at each time step, whereas FFANNs do not need to do so, as they do not share parameters across each layer (CNNs do share weights too, but such sharing occurs through the feature space and not through time).

There are several variants in the RNN reign. For instance, Bidirectional Recurrent Neural Networks (BRNNs) pull information from future data in order to improve the accuracy and LSTM and Gated Recurrent Unit (GRU) are created as a solution to the vanishing gradient problem.^99^ Recently, attention mechanisms have been introduced in new algorithms configuring the state-of-the-art today. Attention is a technique that mimics the cognitive attention process in the human brain. Initially, it was applied to solve typical problems normally tackled by RNNs but completely precluding recurrence. Today, attention almost completely spans the ML application landscape. Attention enhances the more relevant parts of the input data while fading out the rest in regard to the task that the network seeks to solve. Famous examples with big breakthroughs are Generative Pretrained Transformer (GPT) 2 and 3,^100,101^ as well as Bidirectional Encoder Representations from Transformers (BERT).^102^

## How To Get Started

**Figure 5:**
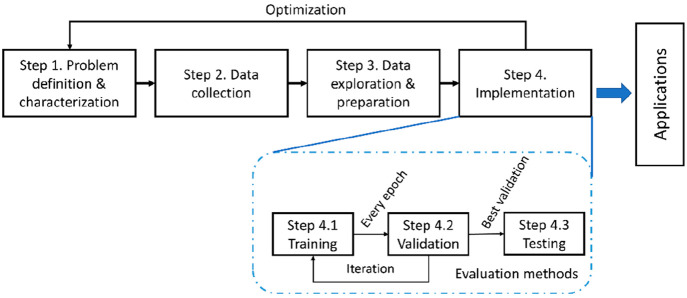
Flowchart of the general steps for building an ML algorithm.

There is a series of feasible steps one could follow to aim for a successful ML application. Yet, such steps will not necessarily be conducted once on the ML process. Conversely, this step-by-step procedure is cyclic, returning once again to the first step and optimizing strategies in each step to achieve better results,^103^ as shown in Figure 5.

First of all, the problem to be solved needs to be characterized. Characterizing a problem means to understand, define, and delineate it by identifying its challenging aspects. Characterizing a problem in ML is to define what the algorithm will have as input information and what it will need to return as output. The loss functions and performance evaluations are selected once the input–output is defined. This is the step where valuable knowledge from domain experts helps in collecting the relevant data in order to understand the target requirements.

The second step is to collect appropriate data sets for training. The volume, type, and quality of the data depend on both the complexity of the ML strategy and the problem defined in Step 1. Typical questions that can arise in this stage include: Is this a classification or regression problem? Is there enough labeled data? Could we approach the problem by generating artificial data to train the model? Can we transfer knowledge from an available data set to a target data? In the case of data scarcity, can we augment our data set? In which way? The NVIDIA Data Loading Library (DALI) is a platform for data loading and preprocessing to accelerate deep learning applications. Using DALI, one can augment their own data sets by offloading them on graphic processing units so as to avoid the bottlenecks of central processing unit in the processing pipeline. Collecting an appropriate data set is a fascinating and complex problem in itself. This problem frequently entails complete investigations in which prestigious research groups devote years of laborious work.^104^

Data exploration and preparation is the third step. On one hand, interacting with the data set, substantially exploring it before its final utilization, is mandatory in order to get more insights that help in the ML strategy selection and optimization. Data exploration includes testing its separability, linearity, monotonicity, and balance, finding its statistical distribution and spatial and/or temporal dependency, etc. On the other hand, data preparation deals with arranging the data for training, validation, and testing procedures. This stage usually includes cleaning, normalizing, segmenting, balancing, etc.

The fourth step concerns implementation, i.e., the ML strategy is selected and then trained and validated to finally be tested. Choosing an appropriate strategy depends on the combination of a multitude of factors such as the kind of data set to be processed and the problem to be solved. A myriad of different architectures can be chosen for different problems. For instance, when the problem is related to computer vision, a suitable architecture is the one with considerable visual inductive bias such as the variant of a CNN. Proper architectures for NLP are the ones with a recurrent structure, such as LSTM or GRU. Nevertheless, all kinds of rules in these aspects have shown to become obsolete with time. Today, the best architectures for NLP are shown to dispense with recurrence using self-attention with transformers.^105^ Likewise, such architectures have been taken from the NLP world and successfully been applied to image classification.^106^ In some cases, a combination, using a self-attentional architecture by preprocessing the inputs using a pretrained CNN as a backbone architecture, is applicable to more complex tasks, such as object detection in computer vision.^107^ However, there is no universally superior ML algorithm according to the no free lunch theorem for ML. Typical DL frameworks are Tensorflow and Pytorch, among others. Choosing the right DL framework for one’s needs is a topic in itself and is beyond the scope of this guide.

After each training epoch, validation is conducted. Validation metrics are chosen to select the best performing epoch in an iterative manner. The current epoch with outperformed results will be stored. Otherwise, it will be discarded. Until the performance meets the requirement, the best one will be used on the testing data set. The validation metrics could be different from or the same as the ones used for the final testing.

For implementation, there are diverse ways to develop and share codes. The most widely used platform is GitHub that is a provider of Internet hosting for software development and version control using Git. Git is the software for tracking changes in any set of files, but it is mostly used for tracking changes in software development files. For sharing data sets and code, general-purpose open-access repositories, such as Zenodo, are the preferred options.

## Properties of ML-Based Algorithms

### Algorithm Performance Evaluation and Benchmark

Performance evaluation is crucial for the development of any algorithm. It directly affects algorithm selection and parameter tuning. Different schemes exist to evaluate the deviation between the ground truth and the prediction generated by an algorithm, known as the error. During training, weights are adjusted to minimize the errors produced on training data sets, i.e., training errors. To evaluate the generalization capacity of the algorithms, the errors produced on the validation data sets, i.e., generalization errors, are relevant for practical applications. Usually, the performance on validation data sets is used during training to select the best performing implemetations. Such implementations will be finally utilized in real *test* data sets. Validation also provides a reference to tune the structural parameters, such as the number of layers and nodes in an NN. Accordingly, by comparing the performance of different algorithms on a validation data set, the most suitable algorithms can be selected for further application in *real-life* scenarios (test data sets).

Regarding the continuous output from the regression tasks, such as the denoised current trace from step 1, extracted spike segments from step 2, continuously varied spike features from step 3, and inferred properties of analytes with continuous values from step 4, relative error (err_r_) and mean-squared error (err_ms_) are usually employed as indexes to evaluate the performance, defined as
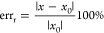
1
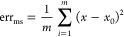
2where *x* is the measured value, *x*_0_ the ground truth, and *m* the total number of output points. The relative errors can be calculated for each data point/situation, and the average and standard deviation of these relative errors on the total output points *m* can be further derived to reflect the overall performance of the algorithm on a certain data set.^35^

For discrete outputs from the classification tasks, such as identified classes of the analytes from step 4 and extracted spike features in qualitative, categorical, or attribute variables, error rate (ER), and accuracy (Acc) are commonly adopted to count the incorrectly and correctly classified data, respectively
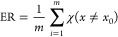
3
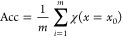
4where χ(.) is the indicator function equal to 1 when the condition is valid and to 0 otherwise. In addition, a standard *F*-measure is widely employed to evaluate the classification performance.^69,83,87^ By comparing with the ground truth, a table named confusion matrix can be derived by evaluating the true positive (TP), false positive (FP), true negative (TN), and false negative (FN) predictions for each class. Two metrics, precision and recall, are defined as
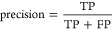
5And
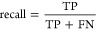
6Precision and recall are not useful when used in isolation. For instance, it is possible to have perfect recall by simply producing a less restrictive classification of samples without worrying about false positives. Similarly, it is also possible to obtain a very high precision by just being very restrictive about the classification of positive samples, virtually invalidating the chances of false positives. Therefrom, the *F* score is a kind of trade-off that combines precision and recall as a figure of merit to evaluate the overall performance, i.e.,
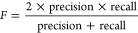
7Furthermore, other performance measures can be commonly seen according to the specific situations, such as receiver operating characteristics and cost curves.^108,109^

To compare the overall performance among various algorithms on a higher level, directly ranking the value of the aforementioned performance indices is not a comprehensive manner. Considering the stochastic factors in the training/test-data selection and training process, hypothesis tests based on the statistical theory are usually adopted.^110^

In general, acquisition of the ground truth is always difficult in performance evaluation. For example, it is usually impossible to acknowledge the ground truth, e.g., a clean signal without noise, true values of amplitude, duration, and FTE, etc., from the measured experimental data. An indirect route is to analyze the rationality and consistency of the outputs with the assistance of related physical models. Apart from experimental results, it can be beneficial to use artificially generated data, if they are accessible, to evaluate an algorithm. Usually, the generated data may come from simulations or modeling from which the ground truth is “known”. Thus, a well-established set of physical models and related simulation frameworks are crucial for evaluating algorithms.

### Reproducibility as a Means for Result Reliability

Reproducibility is inextricably associated with the scientific method in itself. Any result, whether for experimental measurement or algorithm implementation, must be accompanied by clear descriptions delineating its replication procedure under explicit conditions.

In order to obtain reliable results from the signal processing algorithms, a general agreement of experimental data repeatability with considerable signal-to-noise ratio is necessary. Various algorithms have been specifically designed for unique patterns of signals. If the processed signals are closer to such typical cases, the output results are more reliable and interpretative. It is obvious that in the ML-based algorithms, the features/patterns/properties learned are based on the training data sets and regarded as the essential connotations of a certain category distinguished from other categories. Therefore, these acquired connotations should be repeatable and reliable such that they can represent the essential differences among these categories in the real world.

Efforts can be made from two aspects to reinforce the reliability. One is a strict control of experimental conditions to guarantee repeatability as much as it could attain, such as standardization of experimental procedures, careful handling of nanopore devices, robust screening of noise interference, etc. The other is an improvement of the generalization ability of algorithms. Multiple variations should be involved in the data sets for algorithm training toward complicated scenarios so as to boost the robustness. Moreover, a suitable architecture of algorithms with a proper scale should be carefully selected to avoid overfitting due to randomly appearing details.

As for the implementation of ML, any one should be able to achieve the same results by using the code and data. In this way, the same computations could be easily executed to replicate identical results. Nowadays, there are exceptional tools to achieve this endeavor. For instance, a complete project can be shared online using Git and GitHub. A release of the code can be issued at the moment of publication of the experiments. Such a release allows researchers to access the same version of the code in its state at the date of publication. Researchers can also combine such tools with general-purpose open-access repositories, such as Zenodo, that allow for deposition of data sets and research software fully available online under specific licensing conditions.

Yet, today’s advantages regarding ML reproducibility do not end there. Incorporating additional improvements to already cloned implementations is easily achievable to build up on stable releases. The DL frameworks allow the research community to build powerful ML implementations progressively, step by step, through well tested and appropriately optimized baselines. Typical examples of these frameworks include Pytorch and Tensorflow. Such tools enable the research community to coherently build DL applications that can be rapidly modified by reconfiguring hyper parameters and computation graphs in a modularized fashion. With these tools at hand, researchers can not only replicate but also build upon released implementations by modifying them for their own needs.

### Data Preparation and Data Utilization Strategies for Algorithm Training

It is not necessary to show a human infant a giraffe more than twice in order to get her to identify it in different positions and light conditions. Such a scenario is a far-reaching one for ML. Instead, the success of any ML application is at best uncertain without massive amounts of data. For today’s ML standards, data, in considerable amounts, is available for only a subset of powerful companies, and academia is usually left out. Generally, data is considered a scarce resource, let alone accurately labeled data that is far from abundant.^111^ Data has to be annotated manually according to human judgment, which is an extremely costly and time-consuming process. Crowdsourcing, on the other hand, is an alternative approach, which exploits *the crowd* to annotate data and thus significantly reduces human labor and therefore cost. Yet, results from crowdsourcing are far from perfect and bear numerous low-quality annotations.^112^ Appealing to the example of recognizing images, some tasks in this area are simple, such as categorizing dogs, and can be done by nonspecialized staff. Conversely, labeling medical images, such as the ones found in cancerous tissues, needs deep medical expertise, which is extremely hard to access.^113^

Supervised learning is the leading cause for this problematic situation, whereas alternative solutions to this problem can be referred to *nonsupervised paradigms*. For instance, *semisupervised learning* is an extension of supervised learning that uses unlabeled data in conjunction with labeled data to improve learning. Classically, the aim is to obtain enlarged labeled data by assigning labels to unlabeled data using their own predictions.^114^ Another example is *unsupervised representation learning methods* that make use of unlabeled data to learn a representation function *f* such that replacing data point *x* by feature vector *f*(*x*) in new classification tasks reduces the requirement for labeled data. Typical examples of such methods are self-supervised methods and generative algorithms. Finally, *reinforcement learning* is an optimized-data alternative to supervised learning, since the sample complexity does not depend on preexisting data, but rather on the actions that an agent takes in the dynamics of an environment.^115^

Another general solution can be *data augmentation*. It involves a set of methods that apply systematic modifications to the original training data in a way that it creates new samples. It is regularly utilized in classification problems with the aim of reducing the *overfitting* caused by limitations imposed by the size of the training data. Augmentations can be basic or generative, depending on if they are handcrafted by humans or artificially learned by machines via utilizing generative algorithms. They can be applied to data-space or feature-space. They can be supervised or unsupervised depending on if they rely on labels or not.

Knowledge sharing aims at reusing knowledge instead of relying solely on the training data for the main task. This category comprises (i) *transfer learning*, which aims to improve learning and minimize the amount of labeled samples required in a target task by leveraging knowledge from a source task; and (ii) *multitask learning*, which involves no distinction between source, target task, and multiple related tasks. They are learned jointly using a shared representation, (iii) *lifelong learning*, which aims to avoid “catastrophic forgetting” (catastrophic forgetting basically means the loss or disruption of previously learned knowledge when a new task is learned) and (iv) *meta-learning*, which automates the experiments that are required to find the best performing algorithm and parameters of the algorithm resulting in better predictions in shorter time.

In the realm of nanopore translocation events, several applications, such as spike recognition, feature extraction, and analyte identification, can be solved using shallow ML or sophisticated DL schemes. In contrast to traditional algorithms that usually rely on expert knowledge and experience, ML has advanced with important achievements exemplified by its success in addressing most of the issues in this area during the past decades. Shallow ML has mainly offered new mechanisms with the capacity to automatically learn from data, solving problems of feature extraction, classification, identification, and regression, in the signal processing for nanopore sensors. Even when the parameters of the ML algorithms are automatically adjusted from data, the inputs to such algorithms have to be preprocessed considerably in order to make them digestible by the algorithms. However, this preprocessing step usually requires human expertise, which is subjective and sometimes incompatible with the ultimate goal of the learning algorithms.

To overcome this challenge, DL can recognize and automatically extract highly specialized features from the raw data by a training process that agrees with the latest classification or regression stages. This solution can considerably improve the performance of system tasks. By taking such a strategy, DL has been applied to solve analyte classification with automatically extracted features,^70,83^ translocation waveform regression and identification,^25,77^ and noise recognition and elimination^62,87^ in nanopore sensing. Yet, DL has its own drawbacks that render it difficult to implement in some scenarios. To begin with, DL is inherently a data-hungry strategy lacking mechanisms for learning relevant abstractions from few explicitly exposed examples. This pitfall is far from how humans solve problems on a daily basis. Additionally, DL works best when there are thousands, millions, or even billions of training examples.^101^ In problems with limited data sources, DL is not an ideal solution. In the specific area of nanopore sensing, real traces collected from nanopore translocation experiments could be abundant, but they are not labeled. Recruiting staff for labeling such data is not viable, given the extension of the data sets needed to train any conceivable DL architecture. Palliative strategies as the ones discussed above could solve the problem at least partially. For instance, data can be augmented in several ways, knowledge of the system can be transferred to new tasks, and alternative unsupervised tasks can serve as pretraining examples to improve the performance obtained from scarcely labeled data sets. Moreover, it is paramount to develop good strategies to augment the available data or to pretrain the architectures on, for instance, unsupervised tasks before training (fine-tuning) them on labeled data for the final downstream tasks. Generating artificial nanopore translocation signal traces appears to be a good option. Such a path has its own caveats though, since generating a data set with the same probability distribution as the experimental data is an impossible endeavor.

Nonetheless, an approximation can be achieved and the better it is, the better the network can be trained regarding experimental data sets. From the perspective of the architecture, DL offers a rich repertoire of alternatives with different characteristics. A reasonable strategy seems to be following the trends utilized in current state-of-the-art computer vision and language models. For example, it is a reasonable path that employs CNNs as preprocessing backbones pretrained on unsupervised tasks. Afterward, it fine-tunes such backbones using attentional architectures, such as transformers. Finally, it trains them on supervised downstream tasks with reduced labeled data sets. Therefore, it demands new ways of augmenting nanopore translocation data or alternatively generating unsupervised tasks in order to pretrain the architectures.

## Conclusion and Outlook

Nanopore-based sensors have found myriad existing applications and confer potential in a wide range of scientific disciplines and technological areas. The realization of nanopore sensors has critically benefitted from today’s mature biotechnology and semiconductor fabrication technology. Signal processing is an inseparable component of sensing in order to identify the hidden features in the signals and to analyze them. In general, the signal processing flow can be divided into four steps: denoising, spike recognition, feature extraction, and analyte identification. Following this processing flow, the developmental tactics and features of the algorithms at each step are discussed with implementation examples, by categorizing them into ML-based and non-ML-based classes. With the application of ML, the performance of an algorithm is enhanced to a great extent, especially for classification tasks, thus facilitating the wide spectrum of real-life applications of nanopore sensors. Lately, an increasing number of novel algorithms are developed periodically. Thus, in this work, a comprehensive guide is provided with further discussion on the special properties of ML-based algorithms that are shaping up a new paradigm in the field.

A successful nanopore technology builds on two hand-in-hand pillars, i.e., the “hardware” comprising, apart from essential biochemistry, device fabrication, integration, upscaling, electronics, surface management, and the “software” named signal processing. Nanopore sensing signals are generally different from those of other sensing approaches and require special treatments. Three sets of major challenges need to be resolved in order to take full advantage of the great potentials of nanopore technology. (i) The complicated physics in the intertwined processes of ion transport and analyte translocation makes the mechanisms behind signal generation intriguing, since they depend on a large range of different factors. (ii) The nanoconfined space, surface-dominant processes, multiorigin noise, high environmental susceptibility, and weak long-term stability invite serious concerns about the quality of signals. Achieving a quantitative and precise description of the signals can render a challenging proposition. (iii) The great variability found in the configuration of sensor structures and experimental measurements demands the handling of the nanopore signals to seek interpretation of the widely varying data and to standardize procedures, tools, and protocols. In order to respond to these three challenges, two aspects are considered. On one hand, sophisticated physical models based on the established translocation mechanisms are required to assist the evolution of corresponding algorithms. On the other hand, strategies for performance enhancement regarding accuracy, objectivity, robustness, and adaptiveness need be outlined.

As can be seen from the general flow of signal processing for nanopore sensors, each step has its own purpose. No single algorithm/strategy can resolve all problems covering the entire flow. Moreover, this flow is not strict and can be redesigned to take into consideration variations in sensor structures, measurement configurations, target analytes, and application scenarios. Thus, the algorithms are highly application-specific, and some may skip certain steps while others may need to integrate several.

Further development of algorithms for nanopore sensors should consider three aspects. (1) Modularity in each step is a necessity in order to retain the flexibility of the signal processing flow. Users should be able to select suitable algorithms, according to the nature of the data, so as to accomplish the entire task from raw data to final extraction of analyte properties. Standardization of the inputs and outputs is required for each step. (2) Tailorability is another important feature that users should be provided with. Some system parameters for each algorithm, such as format of data, option of data pretreatment, interested feature of the signal, etc., may need reconfiguration in order to adapt to the specified application. (3) A synthetical platform as a package solution is welcome. It integrates several algorithms in all steps and assembles a pipeline of the signal processing by the users’ preference. Furthermore, the performance of different algorithms can be compared systematically, which offers a reference for users’ selection.

Advanced algorithms should be able to assess more stereoscopic data than generated electrically by analyte translocations. Optical and nanomechanical signals are complementary examples. Algorithms of the next level are also able to evaluate experiment-related information such as design and fabrication parameters and characterization conditions. Co-design of experiment and algorithm would be the ultimate.

## Vocabulary

Nanopore sensor: A sensor device with a nanoscale pore in a separation membrane. It usually works in electrolytes to count or analyze analytes, such as biomolecules, upon their passage through the pore that generates signals of electrical, optical, or mechanical nature
Pulse-like signal: A time-sequence signal that consists of rapidly increasing-decreasing changes, i.e., pulses, on a generally stable baseline. The appearance of these pulses can be either random, such as neuron spike signals and nanopore translocation signals, or regular, such as electrocardiographic signals
Machine learning: A family of algorithms whose current output is associated with its history input or the distribution of the history input. Hence, such algorithms are built on recurrently “learning” from the history/distribution of the input. Such learning can be either explicit, as in a supervised training process, or implicit, as in some unsupervised clustering algorithms
Signal features: Abstract parameters that characterize the information of a signal. Feature extraction is a process of information compression that uses several parameters to represent the information of interest for the signal
Labeled data: Data with correct answers. They are marked and subsequently used for training an algorithm in accordance to a supervised learning process. These correct answers are the information of interest that are anticipated from the algorithm upon inputting corresponding data. They can be the right class of the data and/or the right values of certain quantities of the data
Deep vs. shallow learning: Two different categories of machine learning algorithms. Deep learning is based on a huge number of tunable parameters that can be even larger than the amount of training data, such as those in a neural network, while shallow leaning uses a relatively smaller scale of tunable parameters, such as those in a support vector machine
Classification vs regression: Two distinct categories of machine learning tasks. A classification task requires that an algorithm identify the input data and divide them into several preset classes, while a regression task anticipates that an algorithm predict the values of continuous variables, usually the characteristic quantities of the data

## References

[ref1] Deamer D.; Akeson M.; Branton D. Three Decades of Nanopore Sequencing. Nat. Biotechnol. 2016, 34, 518–524. 10.1038/nbt.3423. 27153285 PMC6733523

[ref2] Luo Y.; Wu L.; Tu J.; Lu Z. Application of Solid-State Nanopore in Protein Detection. Int. J. Mol. Sci. 2020, 21, 2808 10.3390/ijms21082808. PMC7215903 32316558

[ref3] Wu H.-C.; Astier Y.; Maglia G.; Mikhailova E.; Bayley H. Protein Nanopores with Covalently Attached Molecular Adapters. J. Am. Chem. Soc. 2007, 129, 16142–16148. 10.1021/ja0761840. 18047341

[ref4] Borsley S.; Cockroft S. L. *In Situ* Synthetic Functionalization of a Transmembrane Protein Nanopore. ACS Nano 2018, 12, 786–794. 10.1021/acsnano.7b08105. 29244946

[ref5] Goyal G.; Freedman K. J.; Kim M. J. Gold Nanoparticle Translocation Dynamics and Electrical Detection of Single Particle Diffusion Using Solid-State Nanopores. Anal. Chem. 2013, 85, 8180–8187. 10.1021/ac4012045. 23885645

[ref6] Venta K. E.; Zanjani M. B.; Ye X.; Danda G.; Murray C. B.; Lukes J. R.; Drndic M. Gold Nanorod Translocations and Charge Measurement through Solid-State Nanopores. Nano Lett. 2014, 14, 5358–5364. 10.1021/nl502448s. 25093657

[ref7] Rhee M.; Burns M. A. Nanopore Sequencing Technology: Research Trends and Applications. Trends Biotechnol. 2006, 24, 580–586. 10.1016/j.tibtech.2006.10.005. 17055093

[ref8] O’Donnell C. R.; Wiberg D. M.; Dunbar W. B. A Kalman Filter for Estimating Nanopore Channel Conductance in Voltage-Varying Experiments. IEEE 51st Conference on Decision and Control (IEEE CDC) 2012, 2304–2309. 10.1109/CDC.2012.6426129.

[ref9] Raillon C.; Granjon P.; Graf M.; Steinbock L.; Radenovic A. Fast and Automatic Processing of Multi-Level Events in Nanopore Translocation Experiments. Nanoscale 2012, 4, 4916–4924. 10.1039/c2nr30951c. 22786690

[ref10] Albrecht T.; Slabaugh G.; Alonso E.; Al-Arif S. M. R. Deep Learning for Single-Molecule Science. Nanotechnology 2017, 28, 423001 10.1088/1361-6528/aa8334. 28762339

[ref11] Das N.; Mandal N.; Sekhar P. K.; RoyChaudhuri C. Signal Processing for Single Biomolecule Identification Using Nanopores: A Review. IEEE Sens. J. 2021, 21, 12808–11820. 10.1109/JSEN.2020.3032451.

[ref12] Cui F.; Yue Y.; Zhang Y.; Zhang Z.; Zhou H. S. Advancing Biosensors with Machine Learning. ACS Sens. 2020, 5, 3346–3364. 10.1021/acssensors.0c01424. 33185417

[ref13] Taniguchi M. Combination of Single-Molecule Electrical Measurements and Machine Learning for the Identification of Single Biomolecules. ACS Omega 2020, 5, 959–964. 10.1021/acsomega.9b03660. 31984250 PMC6977028

[ref14] Arima A.; Tsutsui M.; Washio T.; Baba Y.; Kawai T. Solid-State Nanopore Platform Integrated with Machine Learning for Digital Diagnosis of Virus Infection. Anal. Chem. 2021, 93, 215–227. 10.1021/acs.analchem.0c04353. 33251802

[ref15] Ma H.; Ying Y.-L. Recent Progress on Nanopore Electrochemistry and Advanced Data Processing. Curr. Opin. Electrochem. 2021, 26, 100675 10.1016/j.coelec.2020.100675.

[ref16] Eggenberger O. M.; Ying C.; Mayer M. Surface Coatings for Solid-State Nanopores. Nanoscale 2019, 11, 19636–19657. 10.1039/C9NR05367K. 31603455

[ref17] Wang J.; Bafna J. A.; Bhamidimarri S. P.; Winterhalter M. Small-Molecule Permeation across Membrane Channels: Chemical Modification to Quantify Transport across OmpF. Angew. Chem., Int. Ed. 2019, 58, 4737–4741. 10.1002/anie.201814489. 30701680

[ref18] Wei R.; Gatterdam V.; Wieneke R.; Tampé R.; Rant U. Stochastic Sensing of Proteins with Receptor-Modified Solid-State Nanopores. Nat. Nanotechnol. 2012, 7, 257–263. 10.1038/nnano.2012.24. 22406921

[ref19] Yusko E. C.; Johnson J. M.; Majd S.; Prangkio P.; Rollings R. C.; Li J.; Yang J.; Mayer M. Controlling Protein Translocation through Nanopores with Bio-Inspired Fluid Walls. Nat. Nanotechnol. 2011, 6, 253–260. 10.1038/nnano.2011.12. 21336266 PMC3071889

[ref20] Wang H.-Y.; Song Z.-Y.; Zhang H.-S.; Chen S.-P. Single-Molecule Analysis of Lead(II)-Binding Aptamer Conformational Changes in an α-hemolysin Nanopore, and Sensitive Detection of Lead(II). Microchim. Acta 2016, 183, 1003–1010. 10.1007/s00604-015-1699-x.

[ref21] Galenkamp N. S.; Soskine M.; Hermans J.; Wloka Z.; Maglia G. Direct Electrical Quantification of Glucose and Asparagine from Bodily Fluids Using Nanopores. Nat. Commun. 2018, 9, 4085 10.1038/s41467-018-06534-1. 30291230 PMC6173770

[ref22] Duda R. O.; Hart P. E. Pattern Classification and Scene Analysis, 2nd ed.; John Wiley & Sons: New York, 2001.

[ref23] Zhao Y.; Ashcroft B.; Zhang P.; Liu H.; Sen S.; Song W.; Im J.; Gyarfas B.; Manna S.; Biswas S.; Borges C.; Lindsay S. Single-Molecule Spectroscopy of Amino Acids and Peptides by Recognition Tunnelling. Nat. Nanotechnol. 2014, 9, 466–473. 10.1038/nnano.2014.54. 24705512 PMC4047173

[ref24] Im J.; Biswas S.; Liu H.; Zhao Y.; Sen S.; Biswas S.; Ashcroft B.; Borges C.; Wang X.; Lindsay S.; Zhang P. Electronic Single-Molecule Identification of Carbohydrate Isomers by Recognition Tunnelling. Nat. Commun. 2016, 7, 13868 10.1038/ncomms13868. 28000682 PMC5187581

[ref25] Celik N.; O’Brien F.; Brennan S.; Rainbow R. D.; Dart C.; Zheng Y.; Coenen F.; Barrett-Jolley R. Deep-Channel Uses Deep Neural Networks to Detect Single-Molecule Events from Patch-Clamp Data. Comm. Biol. 2020, 3, 1–10. 10.1038/s42003-019-0729-3. PMC6946689 31925311

[ref26] Wen C.; Zeng S.; Arstila K.; Sajavaara T.; Zhu Y.; Zhang Z.; Zhang S.-L. Generalized Noise Study of Solid-State Nanopores at Low Frequencies. ACS Sens. 2017, 2, 300–307. 10.1021/acssensors.6b00826. 28723146

[ref27] Wen C.; Zhang S.-L. Fundamentals and Potentials of Solid-State Nanopores: A Review. J. Phys. D: Appl. Phys. 2021, 54, 023001 10.1088/1361-6463/ababce.

[ref28] Pedone D.; Firnkes M.; Rant U. Data Analysis of Translocation Events in Nanopore Experiments. Anal. Chem. 2009, 81, 9689–9694. 10.1021/ac901877z. 19877660

[ref29] Shekar S.; Chien C.-C.; Hartel A.; Ong P.; Clarke O. B.; Marks A.; Drndic M.; Shepard K. L. Wavelet Denoising of High-Bandwidth Nanopore and Ion-Channel Signals. Nano Lett. 2019, 19, 1090–1097. 10.1021/acs.nanolett.8b04388. 30601669 PMC6904930

[ref30] Jagtiani A. V.; Sawant R.; Carletta J.; Zhe J. Wavelet Transform-Based Methods for Denoising of Coulter Counter Signals. Meas. Sci. Technol. 2008, 19, 065102 10.1088/0957-0233/19/6/065102.

[ref31] Yan B.; Cui H.; Zhou J.; Wang H. Electrical Noises Reduction in Nanopores Experiments Based on Consensus Filter. Quim. Nova 2020, 43, 837–843. 10.21577/0100-4042.20170560.

[ref32] Forstater J. H.; Briggs K.; Robertson J. W.; Ettedgui J.; Marie-Rose O.; Vaz C.; Kasianowicz J. J.; Tabard-Cossa V.; Balijepalli A. MOSAIC: A Modular Single-Molecule Analysis Interface for Decoding Multistate Nanopore Data. Anal. Chem. 2016, 88, 11900–11907. 10.1021/acs.analchem.6b03725. 27797501 PMC5516951

[ref33] WANG H.-F.; HUANG F.; GU Z.; HU Z.-L.; YING Y.-L.; YAN B.-Y.; LONG Y.-T. Real-Time Event Recognition and Analysis System for Nanopore Study. Chin. J. Anal. Chem. 2018, 46, 843–850. 10.1016/S1872-2040(18)61090-4.

[ref34] Plesa C.; Dekker C. Data Analysis Methods for Solid-State Nanopores. Nanotechnology 2015, 26, 084003 10.1088/0957-4484/26/8/084003. 25648179

[ref35] Dematties D.; Wen C.; Pérez M. D.; Zhou D.; Zhang S.-L. Deep Learning of Nanopore Sensing Signals Using a Bi-Path Network. ACS Nano 2021, 1 10.1021/acsnano.1c03842. 34583465 PMC8482760

[ref36] Zeng S.; Wen C.; Solomon P.; Zhang S.-L.; Zhang Z. Rectification of Protein Translocation in Truncated Pyramidal Nanopores. Nat. Nanotechnol. 2019, 14, 1056–1062. 10.1038/s41565-019-0549-0. 31591525

[ref37] Huang Y.; Magierowski S.; Ghafar-Zadeh E.; Wang C. A High-Speed Real-Time Nanopore Signal Detector. IEEE Conference on Computational Intelligence in Bioinformatics and Computational Biology (IEEE CIBCB) 2015, 1–8. 10.1109/CIBCB.2015.7300316.

[ref38] Talaga D. S.; Li J. Single-Mmolecule Protein Unfolding in Solid State Nanopores. J. Am. Chem. Soc. 2009, 131, 9287–9297. 10.1021/ja901088b. 19530678 PMC2717167

[ref39] Balijepalli A.; Ettedgui J.; Cornio A. T.; Robertson J. W.; Cheung K. P.; Kasianowicz J. J.; Vaz C. Quantifying Short-Lived Events in Multistate Ionic Current Measurements. ACS Nano 2014, 8, 1547–1553. 10.1021/nn405761y. 24397836 PMC3943493

[ref40] Balijepalli A.; Ettedgui J.; Cornio A. T.; Robertson J. W.; Cheung K. P.; Kasianowicz J. J.; Vaz C. Correction to Quantifying Short-Lived Events in Multistate Ionic Channel Measurements. ACS Nano 2015, 9, 12583–12583. 10.1021/acsnano.5b06216. 26571360 PMC4690192

[ref41] Gu Z.; Ying Y.-L.; Cao C.; He P.; Long Y.-T. Accurate Data Process for Nanopore Analysis. Anal. Chem. 2015, 87, 907–913. 10.1021/ac5028758. 25514172

[ref42] Dunbar W. B. Comment on Accurate Data Process for Nanopore Analysis. Anal. Chem. 2015, 87, 10650–10652. 10.1021/acs.analchem.5b02281. 26414231

[ref43] Gu Z.; Ying Y.-L.; Cao C.; He P.; Long Y.-T. Reply to Comment on Accurate Data Process for Nanopore Analysis. Anal. Chem. 2015, 87, 10653–10656. 10.1021/acs.analchem.5b03225. 25514172

[ref44] Zhang N.; Hu Y.-X.; Gu Z.; Ying Y.-L.; He P.-G.; Long Y.-T. An Integrated Software System for Analyzing Nanopore Data. Chin. Sci. Bull. 2014, 59, 4942–4945. 10.1007/s11434-014-0660-4.

[ref45] Loeff L.; Kerssemakers J. W.; Joo C.; Dekker C. AutoStepfinder: A Fast and Auto-Mated Step Detection Method for Single-Molecule Analysis. Patterns 2021, 2, 100256 10.1016/j.patter.2021.100256. 34036291 PMC8134948

[ref46] Kerssemakers J. W.; Munteanu E. L.; Laan L.; Noetzel T. L.; Janson M. E.; Dogterom M. Assembly Dynamics of Microtubules at Molecular Resolution. Nature 2006, 442, 709–712. 10.1038/nature04928. 16799566

[ref47] Gnanasambandam R.; Nielsen M. S.; Nicolai C.; Sachs F.; Hofgaard J. P.; Dreyer J. K. Unsupervised Idealization of Ion Channel Recordings by Minimum Description Length: Application to Human PIEZO1-Channels. Front. Neuroinform. 2017, 11, 31 10.3389/fninf.2017.00031. 28496407 PMC5406404

[ref48] Tsutsui M.; Yoshida T.; Yokota K.; Yasaki H.; Yasui T.; Arima A.; Tonomura W.; Nagashima K.; Yanagida T.; Kaji N.; Taniguchi M.; Washio T.; Baba Y.; Kawai T. Discriminating Single-Bacterial Shape Using Low-Aspect-Ratio Pores. Sci. Rep. 2017, 7, 17371 10.1038/s41598-017-17443-6. 29234023 PMC5727063

[ref49] Arima A.; Harlisa I. H.; Yoshida T.; Tsutsui M.; Tanaka M.; Yokota K.; Tonomura W.; Yasuda J.; Taniguchi M.; Washio T.; Okochi M.; Kawai T. Identifying Single Viruses Using Biorecognition Solid-State Nanopores. J. Am. Chem. Soc. 2018, 140, 16834–16841. 10.1021/jacs.8b10854. 30475615

[ref50] Arima A.; Tsutsui M.; Harlisa I. H.; Yoshida T.; Tanaka M.; Yokota K.; Tonomura W.; Taniguchi M.; Okochi M.; Washio T.; Kawai T. Selective Detections of Single-Viruses Using Solid-State Nanopores. Sci. Rep. 2018, 8, 16305 10.1038/s41598-018-34665-4. 30390013 PMC6214978

[ref51] Im J.; Sen S.; Lindsay S.; Zhang P. Recognition Tunneling of Canonical and Modified RNA Nucleotides for Their Identification with the Aid of Machine Learning. ACS Nano 2018, 12, 7067–7075. 10.1021/acsnano.8b02819. 29932668

[ref52] Larkin J.; Henley R. Y.; Muthukumar M.; Rosenstein J. K.; Wanunu M. High-Bandwidth Protein Analysis Using Solid-State Nanopores. Biophys. J. 2014, 106, 696–704. 10.1016/j.bpj.2013.12.025. 24507610 PMC3944622

[ref53] Sha J.; Si W.; Xu B.; Zhang S.; Li K.; Lin K.; Shi H.; Chen Y. Identification of Spherical and Nonspherical Proteins by a Solid-State Nanopore. Anal. Chem. 2018, 90, 13826–13831. 10.1021/acs.analchem.8b04136. 30406650

[ref54] Tsutsui M.; Yokota K.; Arima A.; He Y.; Kawai T. Solid-State Nanopore Time-of-Flight Mass Spectrometer. ACS Sens. 2019, 4, 2974–2979. 10.1021/acssensors.9b01470. 31576750

[ref55] Houghtaling J.; List J.; Mayer M. Nanopore-Based, Rapid Characterization of Individual Amyloid Particles in Solution: Concepts, Challenges, and Prospects. Small 2018, 14, 1802412 10.1002/smll.201802412. 30225962

[ref56] Lan W.-J.; Holden D. A.; Zhang B.; White H. S. Nanoparticle Transport in Conical-Shaped Nanopores. Anal. Chem. 2011, 83, 3840–3847. 10.1021/ac200312n. 21495727

[ref57] Wei X.; Ma D.; Zhang Z.; Wang L. Y.; Gray J. L.; Zhang L.; Zhu T.; Wang X.; Lenhart B. J.; Yin Y.; Wang Q.; Liu C. N-Terminal Derivatization-Assisted Identification of Individual Amino Acids Using a Biological Nanopore Sensor. ACS Sens. 2020, 5, 1707–1716. 10.1021/acssensors.0c00345. 32403927 PMC7978492

[ref58] Das N.; Ray R.; Ray S.; Roychaudhuri C. Intelligent Quantification of Picomolar Protein Concentration in Serum by Functionalized Nanopores. IEEE Sens. J. 2018, 18, 10183–10191. 10.1109/JSEN.2018.2872853.

[ref59] Houghtaling J.; Ying C.; Eggenberger O. M.; Fennouri A.; Nandivada S.; Acharjee M.; Li J.; Hall A. R.; Mayer M. Estimation of Shape, Volume, and Dipole Moment of Individual Proteins Freely Transiting a Synthetic Nanopore. ACS Nano 2019, 13, 5231–5242. 10.1021/acsnano.8b09555. 30995394

[ref60] Yusko E. C.; Bruhn B. R.; Eggenberger O. M.; Houghtaling J.; Rollings R. C.; Walsh N. C.; Nandivada S.; Pindrus M.; Hall A. R.; Sept D.; Li J.; Kalonia D. S.; Mayer M. Real-Time Shape Approximation and Fingerprinting of Single Proteins Using a Nanopore. Nat. Nanotechnol. 2017, 12, 360–367. 10.1038/nnano.2016.267. 27992411

[ref61] Liu X.; Zeng Q.; Liu C.; Wang L. A Fourier Transform-Induced Data Process for Label-Free Selective Nanopore Analysis under Sinusoidal Voltage Excitations. Anal. Chem. 2020, 92, 11635–11643. 10.1021/acs.analchem.0c01339. 32786474

[ref62] Tsutsui M.; Takaai T.; Yokota K.; Kawai T.; Washio T. Deep Learning-Enhanced Nanopore Sensing of Single-Nanoparticle Translocation Dynamics. Small Methods. 2021, 5, 2100191 10.1002/smtd.202100191. 34928002

[ref63] Schreiber J.; Karplus K. Analysis of Nanopore Data Using Hidden Markov Models. Bioinformatics 2015, 31, 1897–1903. 10.1093/bioinformatics/btv046. 25649617 PMC4553831

[ref64] Landry M.; Winters-Hilt S. Analysis of Nanopore Detector Measurements Using Machine-Learning Methods, with Application to Single-Molecule Kinetic Analysis. BMC Bioinf. 2007, 8, S12 10.1186/1471-2105-8-S7-S12. PMC2099480 18047711

[ref65] Zhang J.-H.; Liu X.-L.; Hu Z.-L.; Ying Y.-L.; Long Y.-T. Intelligent Identification of Multi-Level Nanopore Signatures for Accurate Detection of Cancer Biomarkers. Chem. Commun. 2017, 53, 10176–10179. 10.1039/C7CC04745B. 28852755

[ref66] Zhang J.; Liu X.; Ying Y.-L.; Gu Z.; Meng F.-N.; Long Y.-T. High-Bandwidth Nanopore Data Analysis by Using a Modified Hidden Markov Model. Nanoscale 2017, 9, 3458–3465. 10.1039/C6NR09135K. 28232981

[ref67] Sui X.-J.; Li M.-Y.; Ying Y.-L.; Yan B.-Y.; Wang H.-F.; Zhou J.-L.; Gu Z.; Long Y.-T. Aerolysin Nanopore Identification of Single Nucleotides Using the AdaBoost Model. J. Anal. Test. 2019, 3, 134–139. 10.1007/s41664-019-00088-x.

[ref68] Churbanov A.; Baribault C.; Winters-Hilt S. Duration Learning for Analysis of Nanopore Ionic Current Blockades. BMC Bioinf. 2007, 8, S14 10.1186/1471-2105-8-S7-S14. PMC2099482 18047713

[ref69] Wei Z.-X.; Ying Y.-L.; Li M.-Y.; Yang J.; Zhou J.-L.; Wang H.-F.; Yan B.-Y.; Long Y.-T. Learning Shapelets for Improving Single-Molecule Nanopore Sensing. Anal. Chem. 2019, 91, 10033–10039. 10.1021/acs.analchem.9b01896. 31083925

[ref70] Diaz Carral A.; Ostertag M.; Fyta M. Deep Learning for Nanopore Ionic Current Blockades. J. Chem. Phys. 2021, 154, 044111 10.1063/5.0037938. 33514094

[ref71] Wang L. Support Vector Machines: Theory and Applications; Springer: Berlin Heidelberg, 2005.

[ref72] Konnanath B.; Sattigeri P.; Mathew T.; Spanias A.; Prasad S.; Goryll M.; Thornton T.; Knee P. Acquiring and Classifying Signals from Nanopores and Ion-Channels. Artificial Neural Networks-International Conference on Artificial Neural Networks (ICANN) 2009, 5769, 265–274. 10.1007/978-3-642-04277-5_27.

[ref73] Meyer N.; Janot J.-M.; Lepoitevin M.; Smietana M.; Vasseur J.-J.; Torrent J.; Balme S. Machine Learning to Improve the Sensing of Biomolecules by Conical Track-Etched Nanopore. Biosensors 2020, 10, 140 10.3390/bios10100140. PMC7601669 33028025

[ref74] Im J.; Lindsay S.; Wang X.; Zhang P. Single Molecule Identification and Quantification of Glycosaminoglycans Using Solid-State Nanopores. ACS Nano 2019, 13, 6308–6318. 10.1021/acsnano.9b00618. 31121093

[ref75] Biswas S.; Sen S.; Im J.; Biswas S.; Krstic P.; Ashcroft B.; Borges C.; Zhao Y.; Lindsay S.; Zhang P. Universal Readers Based on Hydrogen Bonding or π-π Stacking for Identification of DNA Nucleotides in Electron Tunnel Junctions. ACS Nano 2016, 10, 11304–11316. 10.1021/acsnano.6b06466. 28024337

[ref76] Reynaud L.; Bouchet-Spinelli A.; Janot J.-M.; Buhot A.; Balme S.; Raillon C. Discrimination of α-Thrombin and γ-Thrombin Using Aptamer-Functionalized Nanopore Sensing. Anal. Chem. 2021, 93, 7889–7897. 10.1021/acs.analchem.1c00461. 34038092

[ref77] Kolmogorov M.; Kennedy E.; Dong Z.; Timp G.; Pevzner P. A. Single-Molecule Protein Identification by Sub-Nanopore Sensors. PLoS Comput. Biol. 2017, 13, e1005356 10.1371/journal.pcbi.1005356. 28486472 PMC5423552

[ref78] Kuncheva L. I.; Rodriguez J. J. An Experimental Study on Rotation Forest Ensembles. Multiple Classifier Systems. International Workshop on Multiple Classifier Systems (MCS) 2007, 4472, 459–468. 10.1007/978-3-540-72523-7_46.

[ref79] Hattori S.; Sekido R.; Leong I. W.; Tsutsui M.; Arima A.; Tanaka M.; Yokota K.; Washio T.; Kawai T.; Okochi M. Machine Learning-Driven Electronic Identifications of Single Pathogenic Bacteria. Sci. Rep. 2020, 10, 15525 10.1038/s41598-020-72508-3. 32968098 PMC7512020

[ref80] Taniguchi M.; Minami S.; Ono C.; Hamajima R.; Morimura A.; Hamaguchi S.; Akeda Y.; Kanai Y.; Kobayashi T.; Kamitani W.; Terada Y.; Suzuki K.; Hatori N.; Yamagishi Y.; Washizu N.; Takei H.; Sakamoto O.; Naono N.; Tatematsu K.; Washio T.; Matsuura Y.; Tomono K. Combining Machine Learning and Nanopore Construction Creates an Artificial Intelligence Nanopore for Coronavirus Detection. Nat. Commun. 2021, 12, 3726 10.1038/s41467-021-24001-2. 34140500 PMC8211865

[ref81] Cardozo N.; Zhang K.; Doroschak K.; Nguyen A.; Siddiqui Z.; Strauss K.; Ceze L.; Nivala J. Multiplexed Direct Detection of Barcoded Protein Reporters on a Nanopore Array. Nat. Biotechnol. 2021, 1 10.1038/s41587-021-01002-6. 34385692 PMC8766897

[ref82] LeCun Y.; Bengio Y.; Hinton G. Deep learning. Nature 2015, 521, 436–444. 10.1038/nature14539. 26017442

[ref83] Misiunas K.; Ermann N.; Keyser U. F. QuipuNet: Convolutional Neural Network for Single-Molecule Nanopore Sensing. Nano Lett. 2018, 18, 4040–4045. 10.1021/acs.nanolett.8b01709. 29845855 PMC6025884

[ref84] Ohayon S.; Girsault A.; Nasser M.; Shen-Orr S.; Meller A. Simulation of Single-Protein Nanopore Sensing Shows Feasibility for Whole-Proteome Identification. PLoS Comput. Biol. 2019, 15, e1007067 10.1371/journal.pcbi.1007067. 31145734 PMC6559672

[ref85] Fu X.; Wan Y.; Li X.; Ying Y.; Long Y. Analysis and Classification of Nanopore Data Based on Feature-Level Multi-Modality. 13th International Congress on Image and Signal Processing, BioMedical Engineering and Informatics (CISP-BMEI) 2020, 692–698. 10.1109/CISP-BMEI51763.2020.9263687.

[ref86] Karawdeniya B. I.; Bandara Y. M. N. D. Y.; Khan A. I.; Chen W. T.; Vu H.-A.; Morshed A.; Suh J.; Duttab P.; Kim M. J. Adeno-Associated Virus Characterization for Cargo Discrimination through Nanopore Responsiveness. Nanoscale 2020, 12, 23721 10.1039/D0NR05605G. 33231239 PMC7735471

[ref87] Taniguchi M.; Ohshiro T.; Komoto Y.; Takaai T.; Yoshida T.; Washio T. High-Precision Single-Molecule Identification Based on Single-Molecule Information within a Noisy Matrix. J. Phys. Chem. C 2019, 123, 15867–15873. 10.1021/acs.jpcc.9b03908.

[ref88] Tsutsui M.; Tanaka M.; Marui T.; Yokota K.; Yoshida T.; Arima A.; Tonomura W.; Taniguchi M.; Washio T.; Okochi M.; Kawai T. Identification of Individual Bacterial Cells through the Intermolecular Interactions with Peptide-Functionalized Solid-State Pores. Anal. Chem. 2018, 90, 1511–1515. 10.1021/acs.analchem.7b04950. 29350898

[ref89] Xia K.; Hagan J. T.; Fu L.; Sheetz B. S.; Bhattacharya S.; Zhang F.; Dwyer J. R.; Linhardt R. J. Synthetic Heparan Sulfate Standards and Machine Learning Facilitate the Development of Solid-State Nanopore Analysis. Proc. Natl. Acad. Sci. U. S. A. 2021, 118, e2022806118 10.1073/pnas.2022806118. 33688052 PMC7980385

[ref90] Barati Farimani A.; Heiranian M.; Aluru N. R. Identification of Amino Acids with Sensitive Nanoporous MoS_2_: Towards Machine Learning-Based Prediction. npj 2D Mater. Appl. 2018, 2, 1–9. 10.1038/s41699-018-0060-8.

[ref91] Goodfellow I.; Bengio Y.; Courville A. Deep Learning; MIT Press: Boston, 2016.

[ref92] Lecun Y.; Bottou L.; Bengio Y.; Haffner P. Gradient-Based Learning Applied to Document Recognition. Proc. IEEE 1998, 86, 2278–2324. 10.1109/5.726791.

[ref93] Krizhevsky A.; Sutskever I.; Hinton G. E. ImageNet Classification with Deep Convolutional Neural Networks. Proceedings of the 25th International Conference on Neural Information Processing Systems 2012, 1097–1105.

[ref94] Simonyan K.; Zisserman A. Very Deep Convolutional Networks for Large-Scale Image Recognition. arXiv, 2015, 1409.1556v6. https://arxiv.org/abs/1409.1556 (accessed Sept 1, 2021).

[ref95] Szegedy C.; Wei Liu; Yangqing Jia; Sermanet P.; Reed S.; Anguelov D.; Erhan D.; Vanhoucke V.; Rabinovich A. Going Deeper with Convolutions. 2015 IEEE Conference on Computer Vision and Pattern Recognition (CVPR) 2015, 1 10.1109/CVPR.2015.7298594.

[ref96] Xie S.; Girshick R.; Dollár P.; Tu Z.; He K. Aggregated Residual Transformations for Deep Neural Networks. 2017 IEEE Conference on Computer Vision and Pattern Recognition (CVPR) 2017, 5987 10.1109/CVPR.2017.634.

[ref97] Huang G.; Liu Z.; van der Maaten L.; Weinberger K. Q. Densely Connected Convolutional Networks. 2017 IEEE Conference on Computer Vision and Pattern Recognition (CVPR) 2017, 2261 10.1109/CVPR.2017.243.

[ref98] Ahmad A. M.; Ismail S.; Samaon D. F. Recurrent Neural Network with Backpropagation through Time for Speech Recognition. IEEE International Symposium on Communications and Information Technology (IEEE ISCIT) 2004, 98 10.1109/ISCIT.2004.1412458.

[ref99] Hochreiter S.; Schmidhuber J. Long Short-Term Memory. Neural Comput. 1997, 9, 1735–1780. 10.1162/neco.1997.9.8.1735. 9377276

[ref100] Radford A.; Wu J.; Child R.; Luan D.; Amodei D.; Sutskever I. Language Models Are Unsupervised Multitask Learners; 2019 https://paperswithcode.com/paper/language-models-are-unsupervised-multitask (accessed Sept 1, 2021).

[ref101] Brown T. B.; Mann B.; Ryder N.; Subbiah M.; Kaplan J.; Dhariwal P.; Neelakantan A.; Shyam P.; Sastry G.; Askell A.; Agarwal S.; Herbert-Voss A.; Krueger G.; Henighan T.; Child R.; Ramesh A.; Ziegler D. M.; Wu J.; Winter C.; Hesse C.; Chen M.; Sigler E.; Litwin M.; Gray S.; Chess B.; Clark J.; Berner C.; McCandlish S.; Radford A.; Sutskever I.; Amodei D. Language Models Are Few-Shot Learners. arXiv, 2020, 2005.14165v4. https://arxiv.org/abs/2005.14165 (accessed Sept 1, 2021).

[ref102] Devlin J.; Chang M.-W.; Lee K.; Toutanova K. BERT: Pre-Training of Deep Bidirectional Transformers for Language Understanding. arXiv, 2019, 1810.04805v2. https://arxiv.org/abs/1810.04805 (accessed Sept 1, 2021).

[ref103] Jumper J.; Evans R.; Pritzel A.; Green T.; Figurnov M.; Ronneberger O.; Tunyasuvunakool K.; Bates R.; Žídek A.; Potapenko A.; Bridgland A.; Meyer C.; Kohl S. A. A.; Ballard A. J.; Cowie A.; Romera-Paredes B.; Nikolov S.; Jain R.; Adler J.; Back T.; Petersen S.; Reiman D.; Clancy E.; Zielinski M.; Steinegger M.; Pacholska M.; Berghammer T.; Bodenstein S.; Silver D.; Vinyals O.; Senior A. W.; Kavukcuoglu K.; Kohli P.; Hassabis D. Highly Accurate Protein Structure Prediction with AlphaFold. Nature 2021, 596, 583–589. 10.1038/s41586-021-03819-2. 34265844 PMC8371605

[ref104] Open Ended Learning Team. Stooke A.; Mahajan A.; Barros C.; Deck C.; Bauer J.; Sygnowski J.; Trebacz M.; Jaderberg M.; Mathieu M.; McAleese N.; Bradley-Schmieg N.; Wong N.; Porcel N.; Raileanu R.; Hughes-Fitt S.; Dalibard V.; Czarnecki W. M. Open-Ended Learning Leads to Generally Capable Agents. arXiv, 2021, 2107.12808. https://arxiv.org/abs/2107.12808 (accessed Sept 1, 2021).

[ref105] Vaswani A.; Shazeer N.; Parmar N.; Uszkoreit J.; Jones L.; Gomez A. N.; Kaiser L.; Polosukhin I. Attention Is All You Need. arXiv, 2017, 1706.03762. https://arxiv.org/abs/1706.03762v3 (accessed Sept 1, 2021).

[ref106] Dosovitskiy A.; Beyer L.; Kolesnikov A.; Weissenborn D.; Zhai X.; Unterthiner T.; Dehghani M.; Minderer M.; Heigold G.; Gelly S.; Uszkoreit J.; Houlsby N. An Image Is Worth 16 × 16 Words: Transformers for Image Recognition at Scale. arXiv, 2021, 2010.11929. https://arxiv.org/abs/2010.11929v2 (accessed Sept 1, 2021).

[ref107] Carion N.; Massa F.; Synnaeve G.; Usunier N.; Kirillov A.; Zagoruyko S. End-to-End Object Detection with Transformers. arXiv, 2020, 2005.12872. https://arxiv.org/abs/2005.12872 (accessed Sept 1, 2021).

[ref108] Bradley A. P. The Use of the Area Under the ROC Curve in the Evaluation of Machine Learning Algorithms. Pattern Recognit. 1997, 30, 1145–1159. 10.1016/S0031-3203(96)00142-2.

[ref109] Drummond C.; Holte R. C. Cost Curves: An Improved Method for Visualizing Classifier Performance. Mach. Learn. 2006, 65, 95–130. 10.1007/s10994-006-8199-5.

[ref110] Dietterich T. G. Approximate Statistical Tests for Comparing Supervised Classification Learning Algorithms. Neural Comput. 1998, 10, 1895–1923. 10.1162/089976698300017197. 9744903

[ref111] Adadi A. A Survey on Sata-Efficient Algorithms in Big Data Era. J. Big Data 2021, 8, 24 10.1186/s40537-021-00419-9.

[ref112] Zhou Z.-H. A Brief Introduction to Weakly Supervised Learning. Natl. Sci. Rev. 2018, 5, 44–53. 10.1093/nsr/nwx106.

[ref113] Willemink M. J.; Koszek W. A.; Hardell C.; Wu J.; Fleischmann D.; Harvey H.; Folio L. R.; Summers R. M.; Rubin D. L.; Lungren M. P. Preparing Medical Imaging Data for Machine Learning. Radiology 2020, 295, 4–15. 10.1148/radiol.2020192224. 32068507 PMC7104701

[ref114] Triguero I.; Garcia S.; Herrera F. Self-Labeled Techniques for Semi-Supervised Learning: Taxonomy, Software and Empirical Study. Knowl. Inf. Syst. 2015, 42, 245–284. 10.1007/s10115-013-0706-y.

[ref115] Mou W.; Wen Z.; Chen X. On the Sample Complexity of Reinforcement Learning with Policy Space Generalization. arXiv, 2020, 2008.07353v1. https://arxiv.org/abs/2008.07353 (accessed Sept 1, 2021).

